# Long-Term and Heavy Smoking as a Risk Factor for Lumbar Spinal Stenosis: Evidence from a Large-Scale, Nationwide Population-Based Cohort

**DOI:** 10.3390/jcm14217691

**Published:** 2025-10-29

**Authors:** Ji-Hyun Ryu, Ki-Won Kim, Ju-Yeong Kim

**Affiliations:** 1 Department of Orthopedic Surgery, Yeouido St. Mary’s Hospital, College of Medicine, The Catholic University of Korea, Seoul 07345, Republic of Korea; ziggy777@hanmail.net; 2 Department of Orthopedic Surgery, Gyeongsang National University Changwon Hospital, Gyeongsang National University School of Medicine, 11 Samjeongja-ro, Seongsan-gu, Changwon 51472, Republic of Korea

**Keywords:** lumbar spinal stenosis, cigarette smoking, cohort study, pack-years, age, sex

## Abstract

**Background and Objectives:** Lumbar spinal stenosis (LSS) is a leading cause of disability in older adults, but the role of cigarette smoking in its development remains unclear. This study aimed to clarify the association between smoking and the incidence of LSS, with a focus on dose–response relationships and subgroup variations by age and sex. **Methods:** We conducted a nationwide, population-based cohort study using the Korean National Health Insurance Service database. A total of 2,123,268 adults aged ≥ 40 years who underwent health screening in 2009 were followed until LSS diagnosis, death, or 2020. Smoking status, duration, daily consumption, and pack-years were assessed. Cox proportional hazards models with progressive adjustment for demographic, lifestyle, and clinical factors were applied. **Results:** Over a mean follow-up of 8.2 years (17.5 million person-years), 721,909 new cases of LSS were identified. Fully adjusted models showed higher risk in former (HR 1.047; 95% CI, 1.039–1.056) and current smokers (HR 1.052; 95% CI, 1.044–1.060) compared with never smokers. A clear dose–response pattern was observed, with the greatest risk in heavy smokers (≥40 pack-years; HR 1.207; 95% CI, 1.191–1.222). Subgroup analyses indicated stronger associations among adults aged ≥ 65 years and in women. **Conclusions:** Cigarette smoking was independently associated with an increased risk of LSS, with risk increasing according to lifetime exposure. The findings underscore the importance of smoking cessation strategies to reduce the burden of spinal degeneration, especially in older adults and women.

## 1. Introduction

Lumbar spinal stenosis (LSS), a prevalent degenerative disorder of the spine, is characterized by abnormal narrowing of the spinal canal, resulting in compression of the nerve roots or spinal cord. This condition leads to chronic pain, neurological deficits, and functional disability, thereby substantially impairing patients’ quality of life [1,2]. Globally, LSS ranks among the leading causes of disability in older adults, imposing substantial healthcare costs and contributing to productivity loss. The prevalence of symptomatic LSS is estimated at approximately 8–11% in the general population aged over 60 years [3], and this burden is expected to grow rapidly in aging societies. In particular, Korea is experiencing rapid population aging; the proportion of the population aged ≥65 years is projected to exceed 20% by 2025, further amplifying the clinical and economic impact of LSS [4]. This demographic shift underscores the importance of identifying modifiable risk factors to mitigate the future burden of the disease.

The etiology of LSS can be congenital or acquired, with the latter—most often driven by degenerative changes—being more prevalent in older adults. Multiple factors have been implicated in disease onset and progression, including age, abnormal mechanical loading, genetic predisposition, metabolic syndrome, psychological stress, and physical inactivity [5]. Smoking, a well-established and preventable health hazard, is conclusively linked to cardiovascular disease, malignancy, and chronic respiratory disorders [6]. Emerging evidence also indicates that smoking adversely affects the musculoskeletal system, contributing to intervertebral disk degeneration and accelerated LSS [7]. The proposed biological mechanisms include nicotine-induced vasoconstriction, atherosclerotic changes in the spinal microvasculature, impaired collagen synthesis, reduced nutrient diffusion into the intervertebral disks, and chronic inflammation—all of which may exacerbate degenerative processes in the spine [8,9].

Several observational studies have explored the association between smoking and LSS [10]. However, the exact causal relationship remains unclear [11], as most prior investigations have been limited by small sample sizes, non-representative populations, short follow-up periods, and insufficient adjustment for potential confounders. Furthermore, few studies have examined dose–response relationships using detailed smoking exposure metrics—such as duration, intensity, and cumulative pack-years—or assessed whether these associations vary by age or sex. These gaps in the literature hinder robust causal inference and limit the development of targeted prevention strategies.

Given the high prevalence of both smoking and LSS, elucidating their relationship holds substantial clinical and public health relevance. Leveraging the Korean National Health Insurance Service (NHIS) database—which includes virtually the entire adult population and provides comprehensive health screening and claims data—we conducted a large-scale, nationwide, population-based cohort study. The unique strengths of this study include its nationally representative sample, long-term follow-up, precise classification of smoking exposure, and detailed stratified analyses by demographic and clinical characteristics. This study aimed to investigate the association between smoking and the risk of LSS, focusing on dose–response relationships and exploring whether these associations differ by age and sex.

## 2. Methods

### 2.1. Data Source and Study Population

We utilized the health screening and claims data from the Korean National Health Insurance Service (NHIS), a universal, single-payer system operated by the South Korean government. Nearly all Korean citizens are enrolled, and adults aged 40 years or older are eligible for biennial national health screenings. For this study, we included individuals aged ≥ 40 years who underwent health screening in 2009.

The health screening data include lifestyle factors, anthropometric measurements, laboratory test results, and questionnaire responses. The claims data provide diagnostic and treatment records based on the International Classification of Diseases, 10th Revision (ICD-10).

**Figure 1:**
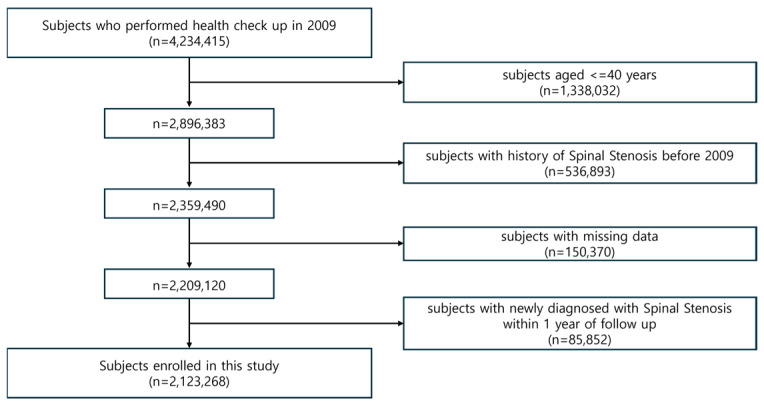
Flow chart of cohort selection.

Initially, 4,234,415 individuals underwent health screening in 2009. After excluding 1,338,032 individuals aged < 40 years, 2,896,383 subjects remained. We further excluded 536,893 individuals with a prior diagnosis of LSS (ICD-10 codes M48.06) before 2009. Another 150,370 individuals with missing data on key variables, including smoking status, were excluded. To minimize the risk of reverse causality, 85,852 participants diagnosed with LSS within one year of their screening date were also excluded. Ultimately, a total of 2,123,268 individuals were included in the final cohort (Figure 1).

Incident lumbar spinal stenosis (LSS) was defined as having at least three separate medical claims with the ICD-10 code M48.06 on different dates. In the Korean National Health Insurance system, a diagnostic code must accompany any reimbursed clinical service, including outpatient consultations, diagnostic imaging, medication prescriptions, spinal injections or procedures, and surgical admissions. Each claim was issued by a board-certified orthopedic or neurosurgical specialist. Therefore, these claims represent comprehensive medical encounters for the diagnosis and treatment of LSS, ensuring high diagnostic validity. This rule was applied throughout the entire post-baseline follow-up without an upper bound on the interval between the first and third claim. This approach captures clinically relevant cases that have been thoroughly evaluated and deemed to require medical attention, rather than isolated radiographic findings. Participants were followed from the date of health screening until the date of LSS diagnosis, death, or 31 December 2020, whichever came first.

### 2.2. Ethics

This study was approved by the Institutional Review Board (IRB) of The Catholic University of Korea. The study was conducted in accordance with the ethical principles of the Declaration of Helsinki. The NHIS database was accessed with official permission, and all data were de-identified. Therefore, informed consent was not required.

### 2.3. Health Screening Variables and Definition of Comorbidities

Health screening data included self-reported lifestyle factors (e.g., smoking, alcohol consumption, physical activity, and income level), anthropometric measurements (e.g., height, weight, waist circumference, and blood pressure), and laboratory tests (e.g., fasting glucose, total cholesterol, HDL-C, LDL-C, and triglycerides). For the purpose of this study, smoking exposure was classified based on self-reported data from the 2009 health screening. Smoking status was categorized as never, former, or current smokers. For former and current smokers, additional variables were collected: smoking duration (in years), number of cigarettes smoked per day, and cumulative smoking exposure (calculated as pack-years). Pack-years were calculated by multiplying the number of packs of cigarettes smoked per day (1 pack = 20 cigarettes) by the number of years the person smoked. These smoking variables, including duration and daily amount, were obtained through standardized self-reported questionnaires administered as part of the NHIS health screening program. These detailed metrics were used to assess dose–response relationships.

Height and weight were measured with shoes off and light clothing, respectively. Body mass index (BMI) was calculated as weight (kg) divided by height squared (m^2^). Blood samples were obtained after at least 8 h of fasting.

Comorbidities were defined as follows:

- **Hypertension**: Presence of ICD-10 codes I10–I13 or I15 along with prescription of antihypertensive medication, or a systolic blood pressure ≥ 140 mmHg or diastolic blood pressure ≥ 90 mmHg at screening.
- **Diabetes mellitus**: Presence of ICD-10 codes E11–E14 along with prescription of antidiabetic medication, or a fasting glucose level ≥ 126 mg/dL.
- **Dyslipidemia**: Presence of ICD-10 code E78, prescription of lipid-lowering agents, or a total cholesterol level ≥ 240 mg/dL.

These definitions were consistent with the prevalence rates presented in the comparison table of smoking status groups. All major comorbidity indicators were confirmed using both anthropometric and biochemical data, ensuring completeness of the dataset.

### 2.4. Statistical Analysis

#### 2.4.1. Basic Statistical Analysis

Baseline characteristics of participants were summarized by smoking status (never, former, and current smokers). Continuous variables were expressed as mean ± standard deviation (SD), and categorical variables as number (n) and percentage (%). Group comparisons were performed using one-way analysis of variance (ANOVA) for continuous variables and the chi-square test for categorical variables. Triglycerides, which did not follow a normal distribution, were reported as median and interquartile range (IQR). All statistical analyses were conducted using two-sided tests, and a *p*-value < 0.05 was considered statistically significant.

The incidence rate (IR) of LSS was calculated as the number of events divided by total person-years of follow-up and presented per 1000 person-years. The association between smoking and the risk of LSS was evaluated using the Cox proportional hazards model. Four models with progressive adjustment for potential confounders were constructed: Model 1: Unadjusted, Model 2: Adjusted for age and sex, Model 3: Model 2 + income level, body mass index (BMI), alcohol consumption, and regular physical activity, Model 4: Model 3 + diabetes mellitus, hypertension, and dyslipidemia. We examined the association between LSS and each smoking exposure variable (smoking status, smoking duration, daily cigarette consumption, and pack-years) using Cox models. We also analyzed combined metrics of smoking status with each exposure (duration, amount, pack-years) to assess whether cumulative smoking burden outweighs current status in determining risk.

In all analyses, never smokers served as the reference group, and the primary interpretation was based on Model 4. Covariate adjustment was prespecified a priori based on clinical and epidemiologic considerations to reduce confounding. Model 4 adjusted for age, sex, household income (quartiles), body mass index, alcohol intake, regular physical activity, diabetes mellitus, hypertension, and dyslipidemia. The covariate set and model sequence were pre-specified before analysis. These variables capture socioeconomic status and major lifestyle/metabolic risk factors that could influence both smoking behavior and LSS risk, and the covariate set (Models 1–4) was pre-specified before analysis.

All statistical analyses were conducted using SAS version 9.4 (SAS Institute, Cary, NC, USA), with two-sided *p*-values and statistical significance set at *p* < 0.05. Visualizations of the results were generated using Python 3.10.18 and the matplotlib library version 3.10.0. The proportional hazards assumption was formally tested for all Cox models using Schoenfeld residuals, and no violations were observed. To evaluate potential multicollinearity among smoking-related variables, variance inflation factors (VIFs) were calculated, all of which were below 5.

#### 2.4.2. Sex- and Age-Stratified Analyses

Subgroup analyses were conducted to assess whether the associations between smoking exposures and the risk of LSS differed by age group and sex.

**Figure A1:**
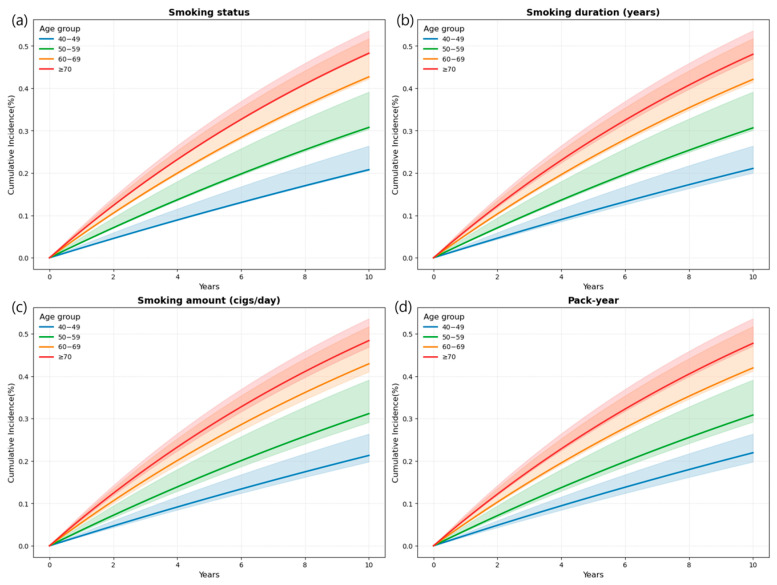
Cumulative incidence of LSS by smoking-related variables, stratified by 10-year age groups. (**a**) Smoking status; (**b**) Smoking duration; (**c**) Smoking amount (cigarettes/day); (**d**) Pack-year. Cumulative incidence curves were estimated over a 10-year follow-up period for each age group (40–49, 50–59, 60–69, and ≥70 years). Shaded areas represent the range of cumulative incidence curves across smoking subgroups for each age group. Note: Numbers at risk at standard time points could not be displayed beneath the curves due to the use of aggregated summary-level data. Instead, group-specific participant numbers and events are provided in Table A2 of Appendix A, which can be cross-referenced for at-risk denominators.

We performed stratified analyses by sex and age to examine effect modification. In addition to using a dichotomized age group (40–64 vs. ≥65 years), we also conducted analyses by finer age strata (in 10-year bands), which showed similar trends (Table A2 and Table A5 and Figure A1 and Figure A3). We likewise stratified the dose–response analyses by age and sex to evaluate differences across demographic subgroups.

For these analyses, Subgroup-specific HRs and 95% CIs were estimated using Cox proportional hazards models (Model 4).

#### 2.4.3. Multivariable Subgroup Analysis

To further investigate potential effect modification by demographic and clinical factors, subgroup analyses were performed. The subgroup variables included age (40–64 vs. ≥65 years), sex, income level (low [Q1] vs. middle-to-high [Q2–Q4]), BMI (≥25 kg/m^2^), abdominal obesity (waist circumference ≥ 90 cm for men, ≥85 cm for women), heavy drinking, regular exercise, diabetes mellitus, hypertension, and dyslipidemia. Within each subgroup, the association between smoking status (former and current vs. never) and risk of LSS was estimated using Model 4. Interaction was assessed by including cross-product terms between smoking status and subgroup variables in the model, and *p*-values for interaction were estimated. All subgroup analyses used the fully adjusted model (Model 4).

## 3. Results

### 3.1. Baseline Characteristics of the Study Population

Among the 2,123,268 participants, 1,306,249 (61.5%) were never smokers, 339,915 (16.0%) were former smokers, and 477,104 (22.5%) were current smokers. All baseline characteristics differed significantly across the three groups (*p* < 0.0001), as summarized in Table 1.

In terms of demographic characteristics, the current smoker group was the youngest, with a mean age of 50.89 ± 8.95 years, compared to 53.38 ± 10.16 years in never smokers and 53.42 ± 9.72 years in former smokers. The proportion of participants aged 40–64 years was highest among current smokers (90.62%). There were substantial differences in sex distribution: the majority of former (96.68%) and current (94.17%) smokers were male, whereas never smokers were predominantly female (72.65%). The proportion of individuals in the lowest income quartile (Q1) was highest in the never smoking group (22.49%) and lowest in the former smoker group (14.7%).

Regarding anthropometric and lifestyle factors, the prevalence of class I obesity (BMI 25.0–29.9) was highest among former smokers (37.85%), who also had the highest mean BMI (24.4 ± 2.8 kg/m^2^) and waist circumference (84.69 ± 7.43 cm). Non-drinking was most prevalent among never smokers (73.05%), whereas heavy drinking was most common among current smokers (18.52%). Regular physical activity was highest in former smokers (27.33%) and lowest in current smokers (18.07%).

For clinical characteristics, the prevalence of diabetes mellitus (13.7%), hypertension (36.77%), and dyslipidemia (22.38%) was highest in the former smoker group. Mean systolic blood pressure (126.19 ± 14.58 mmHg), diastolic blood pressure (78.92 ± 9.93 mmHg), and fasting glucose levels (102.87 ± 27.04 mg/dL) were also highest in this group. Total cholesterol levels were similar across groups: never (198.81), former (197.91), and current (198.61) smokers. HDL-cholesterol was highest in never smokers (57.28 ± 30.43 mg/dL) and lowest in current smokers (52.98 ± 26.26 mg/dL). LDL-cholesterol was lowest in current smokers (112.97 ± 39.36 mg/dL). Triglyceride levels, which were not normally distributed, had the highest median value in current smokers (142.5 mg/dL, IQR: 142.27–142.73), followed by former (129.56 mg/dL) and never smokers (106.45 mg/dL).

These pronounced differences among groups highlight the importance of multivariable adjustment in subsequent analyses.

Current smokers demonstrated greater cumulative smoking exposure (longer duration and higher pack-years) compared to former smokers, which is consistent with their higher prevalence of dyslipidemia and diabetes at baseline.

### 3.2. Association Between Smoking and Risk of LSS

Each analysis was based on population sizes ranging from approximately 1.3 million to 470,000 individuals per smoking category. The number of participants and incident cases by group is presented in Table 2. All counts and rates herein reflect the primary LSS definition of ≥3 M48.06 claims on distinct service dates (index date = third claim).

**Figure 2:**
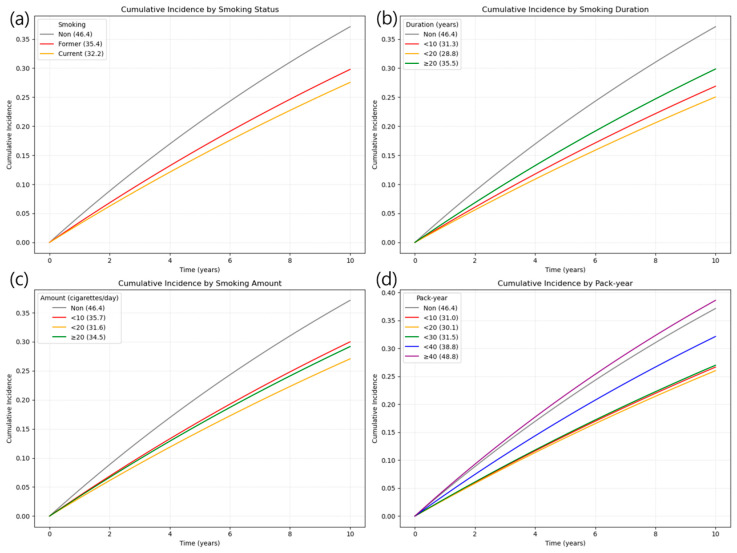
Cumulative incidence of LSS by smoking-related variables over 10 years. (**a**) Smoking status: Highest in never smokers, followed by former and current smokers. (**b**) Smoking duration: ≥20 years showed higher incidence than shorter durations. (**c**) Smoking amount: ≥20 cigarettes/day had the highest incidence among smokers. (**d**) Pack-year: Incidence increased with higher pack-year categories, peaking at ≥40 pack-years. Note: Numbers at risk at standard time points could not be displayed beneath the curves due to the use of aggregated summary-level data. Instead, group-specific participant numbers and events are provided in Table A2 of Appendix A, which can be cross-referenced for at-risk denominators.

**Figure 3:**
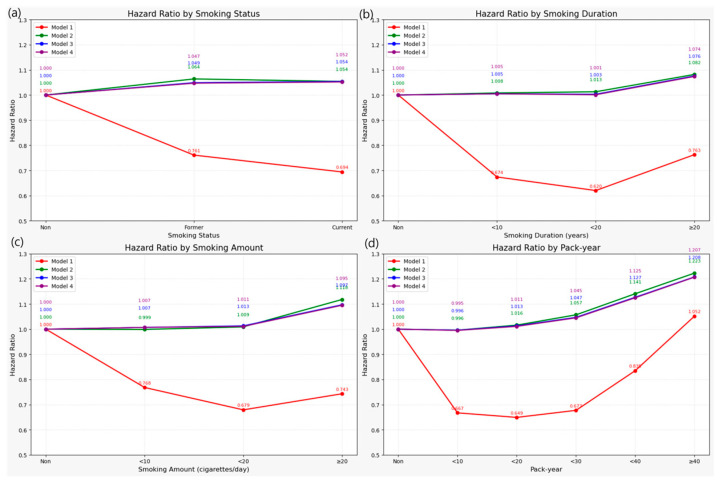
Hazard ratios (HRs) for LSS by smoking-related variables (never smokers as reference, HR = 1.0). (**a**) Smoking status: Fully adjusted HRs were higher in both former and current smokers. (**b**) Smoking duration: Risk increased in the ≥20 years group. (**c**) Smoking amount: ≥20 cigarettes/day had the highest HR among amount categories. (**d**) Pack-year: HRs rose progressively with greater pack-year exposure, highest in ≥40 pack-years.

During a total follow-up period of 17,482,048.38 person-years, 721,909 new cases of LSS were identified (incident LSS required ≥3 M48.06 claims on distinct dates, with the third claim defining the diagnosis date). Although the crude incidence rate of LSS appeared to be the highest among never smokers, this likely reflects confounding due to age and sex composition, as this group was significantly older and more female-dominant. Therefore, these unadjusted rates should be interpreted cautiously and not as evidence of a protective effect of smoking (Figure 2). This counterintuitive pattern is likely attributable to demographic differences, given that the never smoker group was older on average and predominantly female. Across smoking exposure metrics, crude absolute incidence rates (per 1000 person-years) increased with heavier lifetime smoking; for pack-years, IRs ranged from 31.0 in <10 pack-years to 48.8 in ≥40 pack-years (Table 2). Consistently, in the unadjusted analysis (Model 1), current smoking appeared to be associated with a 30.6% lower risk of LSS (HR 0.694; 95% CI, 0.689–0.698) (Figure 3).

After adjustment for age and sex (Model 2), both former (HR 1.064) and current smokers (HR 1.054) showed a significantly increased risk compared to never smokers. These associations persisted in Model 3, which were further adjusted for income level, BMI, alcohol consumption, and physical activity.

In the fully adjusted model (Model 4), which accounted for age, sex, lifestyle factors, and comorbidities, a statistically significant positive association between smoking and incident LSS was observed. Compared with never smokers, the risk was elevated by 4.7% in former smokers (HR 1.047; 95% CI, 1.039–1.056) and 5.2% in current smokers (HR 1.052; 95% CI, 1.044–1.060). Notably, the fully adjusted estimates were close to the age–sex adjusted estimates (Model 2), indicating the limited impact of additional lifestyle and metabolic covariates on the effect size.

A clear dose–response relationship was observed, in which greater cumulative smoking exposure was associated with increased risk. For smoking duration, individuals who had smoked for ≥20 years exhibited a significantly increased risk (HR 1.074; 95% CI, 1.066–1.082), whereas those who smoked for <20 years did not show a significant difference. Daily cigarette consumption showed similar trends: smoking 10–19 cigarettes per day was associated with a modest increase (HR 1.011; 95% CI, 1.003–1.020), while ≥20 cigarettes per day was associated with a notably higher risk (HR 1.095; 95% CI, 1.086–1.104).

The most pronounced dose–response trend was observed for cumulative smoking exposure, measured as pack-years. Risk increased stepwise with higher pack-year categories: HR 1.011 for <20 pack-years, HR 1.045 for 20–29, HR 1.125 for 30–39, and HR 1.207 (95% CI, 1.191–1.222) for ≥40 pack-years. The trend was statistically significant (*p* for trend <0.0001).

#### 3.2.1. Heterogeneity of Smoking Risk by Age

**Figure 4:**
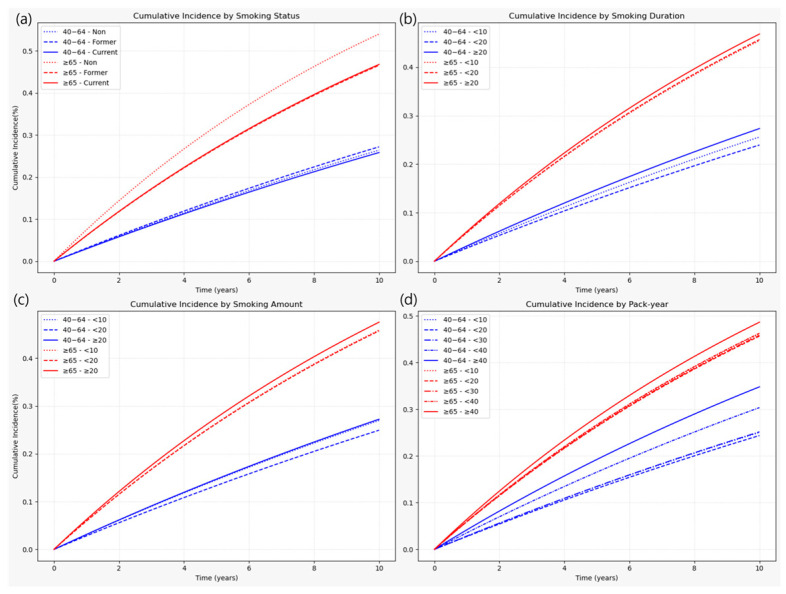
Cumulative incidence of LSS by smoking-related variables, stratified by age groups (40–64 years and ≥65 years). (**a**) Smoking status; (**b**) Smoking duration; (**c**) Smoking amount (cigarettes/day); (**d**) Pack-year. Cumulative incidence curves were estimated over a 10-year follow-up period for two age groups. Different line styles within each panel represent categories of the smoking variable. Note: Numbers at risk at standard time points could not be displayed beneath the curves due to the use of aggregated summary-level data. Instead, group-specific participant numbers and events are provided in Table A1 of Appendix A, which can be cross-referenced for at-risk denominators.

**Figure 5:**
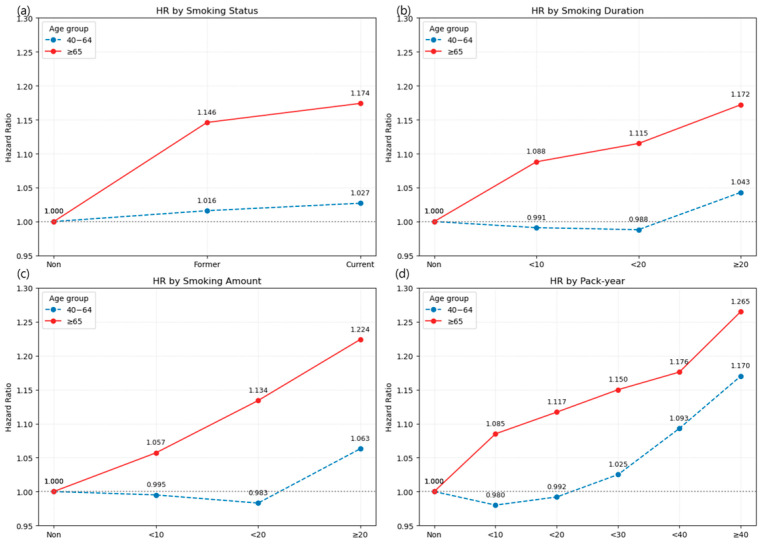
Hazard ratios (HRs) with 95% confidence intervals for LSS by smoking-related variables, stratified by age groups (40–64 years and ≥65 years). (**a**) Smoking status; (**b**) Smoking duration; (**c**) Smoking amount (cigarettes/day); (**d**) Pack-year. HRs were derived from the fully adjusted Cox proportional hazards model (Model 4), adjusting for age, sex, body mass index, income level, alcohol consumption, regular exercise, diabetes mellitus, hypertension, and dyslipidemia. The horizontal dotted line represents HR = 1.0.

The association between smoking and LSS risk varied substantially by age group. In the dichotomized analysis (<65 vs. ≥65 years), both former and current smoking were associated with modest risk increases in the younger group (HR 1.016 and 1.027, respectively), whereas in participants aged ≥ 65 years, the increases were more pronounced (HR 1.146 and 1.174, respectively). Similar patterns were observed for smoking duration, daily cigarette consumption, and pack-years, with stronger associations in older adults. These patterns are visualized in Figure 4 and Figure 5, which show cumulative incidence and hazard ratio plots, respectively, for participants aged 40–64 and those aged ≥ 65 years. Sex- and age-stratified analyses using 10-year age bands (40–49, 50–59, 60–69, and ≥70 years) yielded consistent findings and are presented in Appendix A. Crude absolute incidence rates (IRs per 1000 person-years) were substantially higher in older adults. By smoking status, IRs were 41.9/31.7/29.9 (never/former/current) in ages 40–64 versus 77.6/62.6/63.0 in ages ≥65 (Table A1 of Appendix A). Across exposure metrics, the ≥65 group consistently showed IRs around 60–67, peaking at 66.6 in the ≥40 pack-years category, compared with 28.8–42.8 in ages 40–64 (Table A1 of Appendix A). Stratification by 10-year age bands showed a monotonic rise in non-smoker IRs from 30.6 (40–49 years) to 49.7 (50–59), 72.8 (60–69), and 76.8 (≥70), with parallel increases across exposure categories (Table A2 of Appendix A).

**Figure A2:**
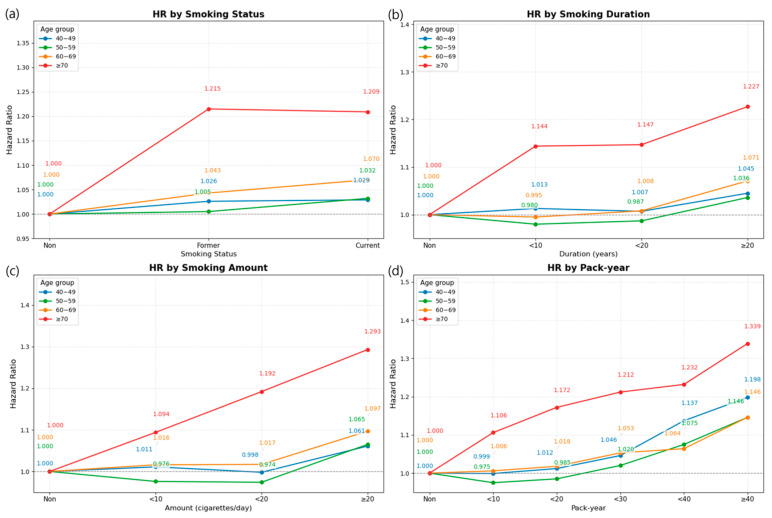
Hazard ratios (HRs) for LSS by smoking-related variables, stratified by 10-year age groups. (**a**) Smoking status; (**b**) Smoking duration; (**c**) Smoking amount (cigarettes/day); (**d**) Pack-year. HRs and 95% confidence intervals were derived from the fully adjusted Cox proportional hazards model (Model 4), adjusting for age, sex, body mass index, income level, alcohol consumption, regular exercise, diabetes mellitus, hypertension, and dyslipidemia. The horizontal dotted line represents HR = 1.0.

Detailed estimates for the dichotomized age analysis are provided in Table A1 of Appendix A, while the results for 10-year age bands are shown in Table A2 of Appendix A and Figure A1 and Figure A2 of Appendix A.

#### 3.2.2. Heterogeneity of Smoking Risk by Sex

**Figure 6:**
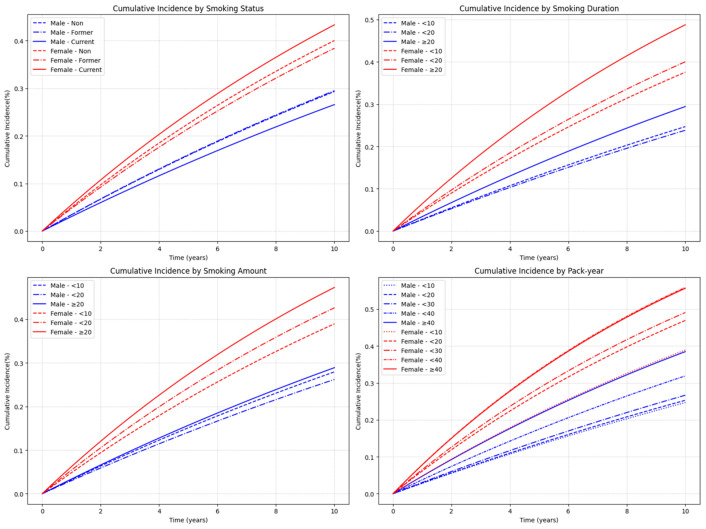
Cumulative incidence of LSS by smoking-related variables, stratified by sex. Male participants are shown in blue and female participants in red, with different line styles representing categories within each smoking variable. Curves were estimated over a 10-year follow-up period. Note: Numbers at risk at standard time points could not be displayed beneath the curves due to the use of aggregated summary-level data. Instead, group-specific participant numbers and events are provided in Table A3 of Appendix A, which can be cross-referenced for at-risk denominators.

**Figure 7:**
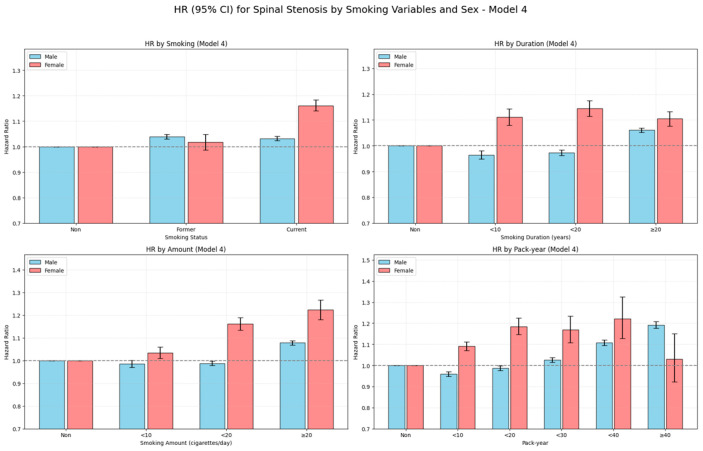
Fully adjusted hazard ratios (Model 4) with 95% confidence intervals for LSS by smoking-related variables, stratified by sex. Adjustments included age, body mass index, income level, alcohol consumption, regular exercise, diabetes mellitus, hypertension, and dyslipidemia. The horizontal dashed line indicates HR = 1.0.

Marked sex differences were observed in the association between smoking and LSS, with women appearing more susceptible to the harmful effects of smoking than men. Among current smokers, women had a substantially greater relative risk increase compared with men. Former smoking was associated with an elevated risk in men but showed no significant association in women. Across all smoking duration, amount, and cumulative exposure categories, women generally exhibited higher risk than men, even at lower exposure levels. These patterns were consistent in both cumulative incidence curves and fully adjusted hazard ratio models (Figure 6 and Figure 7; detailed results in Table A3). Sex-specific crude IRs diverged markedly. In men, IRs were 34.7 (never), 34.9 (former), and 30.9 (current); in women, the corresponding IRs were 51.1, 48.4, and 56.8 (Table A3 of Appendix A). At comparable exposures, women had higher absolute rates—for example, ≥20 pack-years: 48.6 in men vs. 81.3 in women; ≥20 years: 34.9 vs. 66.8; ≥20 cigarettes/day: 34.1 vs. 64.0 (Table A3 of Appendix A).

### 3.3. Combined Effect of Smoking Cessation and Cumulative Smoking Burden

To investigate the long-term effect of cumulative smoking exposure and the impact of smoking cessation, we conducted a composite analysis combining smoking status (former vs. current smokers) with detailed exposure indicators, including smoking duration, daily cigarette consumption, and cumulative pack-years. These composite results are presented in Table 3, which summarizes the adjusted hazard ratios (Model 4) across smoking status and exposure level combinations. Crude absolute incidence rates (per 1000 person-years) for each combined category are also reported in Table 3; for illustration, IRs were 46.4 in never smokers, 42.1 in former smokers with ≥20 pack-years, and 34.6 in current smokers with ≥20 pack-years.

A notable finding was that among individuals with ≥20 pack-years of smoking history, former smokers (HR 1.126, 95% CI 1.113–1.139) exhibited a higher risk of LSS than current smokers (HR 1.097, 95% CI 1.087–1.107). This pattern was consistent in those with ≥20 years of smoking (former: HR 1.091 vs. current: HR 1.065) and in those who had smoked ≥20 cigarettes per day (former: HR 1.097 vs. current: HR 1.092). These findings suggest possible irreversible damage from prolonged smoking or the presence of a “sick quitter effect,” wherein individuals with deteriorating health may be more likely to quit.

Among former smokers, a dose–response relationship by past smoking burden was evident. There was no increased risk for <10 pack-years (HR 0.986), but the risk increased to 2.5% in the 10–19 pack-year group (HR 1.025, 95% CI 1.012–1.039) and 12.6% in the ≥20 pack-year group (HR 1.126, 95% CI 1.113–1.139).

Similar trends were observed for smoking duration and daily amount. Former smokers with ≥20 cigarettes per day had a higher risk (HR 1.097; 95% CI 1.086–1.109), as did those with ≥20 years of smoking (HR 1.091; 95% CI 1.080–1.102), compared to current smokers with equivalent exposure. These results indicate that cumulative exposure history may outweigh current smoking status in determining stenosis risk.

Among current smokers, increased risk was primarily limited to those with heavy cumulative exposure. No significant increase was observed for <20 pack-years, whereas those with ≥20 pack-years showed a 9.7% increased risk (HR 1.097, 95% CI 1.087–1.107).

In summary, the risk of LSS appeared to be more strongly driven by cumulative smoking burden than by current smoking status. Long-term, heavy smoking left substantial residual risk even after cessation.

**Figure 8:**
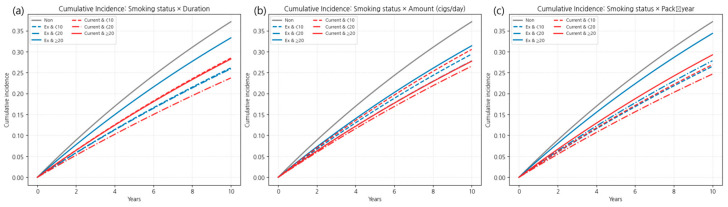
Cumulative incidence of LSS by combined smoking status and exposure level over 10 years. (**a**) Smoking duration; (**b**) Smoking amount (cigarettes/day); (**c**) Pack-year. Former smokers are shown in blue and current smokers in red, with line styles indicating exposure level categories for each variable. Note: Numbers at risk at standard time points could not be displayed beneath the curves due to the use of aggregated summary-level data. Instead, group-specific participant numbers and events are provided in Table 3, which can be cross-referenced for at-risk denominators.

**Figure 9:**
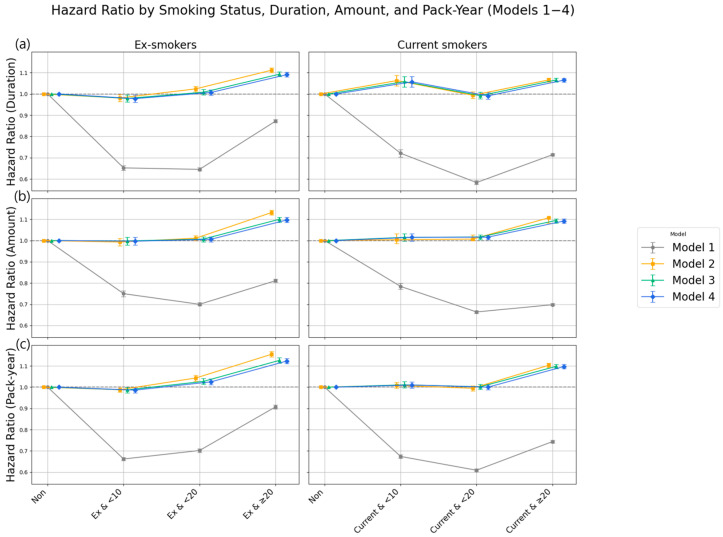
Hazard ratios (HRs) with 95% confidence intervals for LSS by combined smoking status and exposure level (Models 1–4). (**a**) Smoking duration; (**b**) Smoking amount (cigarettes/day); (**c**) Pack-year. HRs were derived from Cox models, with Model 4 fully adjusted for demographic, lifestyle, and comorbidity factors. The horizontal dashed line represents HR = 1.0, and error bars indicate 95% confidence intervals.

Cumulative incidence curves and multivariable-adjusted hazard ratios by smoking status and cumulative exposure are illustrated in Figure 8 and Figure 9, respectively.

#### 3.3.1. Age-Stratified Effects of Combined Smoking Burden and Cessation

**Figure 10:**
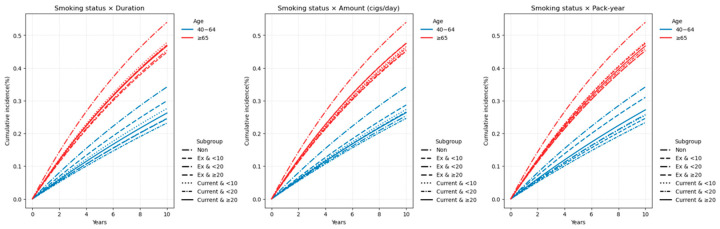
Cumulative incidence of LSS by combined smoking status and cumulative exposure (duration, amount, pack-years), stratified by age group (40–64 years and ≥65 years). In both age groups, higher cumulative exposure was associated with increased incidence, with the steepest curves in older current smokers with heavy exposure. Note: Numbers at risk at standard time points could not be displayed beneath the curves due to the use of aggregated summary-level data. Instead, group-specific participant numbers and events are provided in Table A4 of Appendix B, which can be cross-referenced for at-risk denominators.

**Figure 11:**
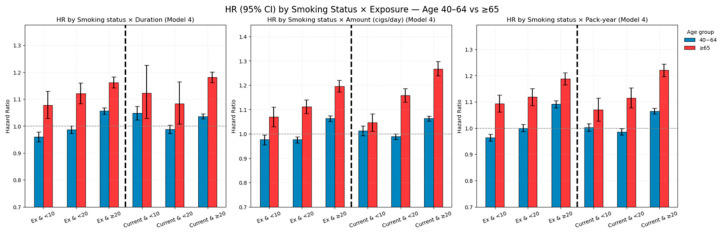
Fully adjusted hazard ratios (Model 4) for LSS by combined smoking status and cumulative exposure, stratified by age group. Risk increased with greater cumulative exposure in both former and current smokers, with consistently higher HRs in the ≥65 years group. Smoking cessation reduced risk but did not fully offset the effects of heavy lifetime exposure.

Age-stratified analysis showed clear heterogeneity in the effects of smoking cessation and cumulative exposure. Detailed results are provided in Table A4 of Appendix B, with corresponding cumulative incidence curves and hazard ratios in Figure 10 and Figure 11. In the combined status–exposure categories, absolute rates were consistently higher in ≥65 years. For current smokers with ≥20 pack-years, the IR was 31.8 in ages 40–64 vs. 63.0 in ≥65; for ≥20 cigarettes/day, 30.6 vs. 64.4; and for ≥20 years of smoking, 30.4 vs. 62.9. Among former smokers with ≥20 pack-years, IRs were 37.2 (40–64) vs. 64.5 (≥65). Baseline non-smoker IRs mirrored this age gap (41.9 vs. 77.6) (Table A4 of Appendix B).

In adults aged 40–64 years, elevated risk was observed mainly in those with high cumulative exposure (≥20 years, ≥20 cigarettes/day, or ≥20 pack-years), regardless of smoking status. Lower exposure levels showed minimal or no significant increase in risk.

In adults aged ≥65 years, the risk was consistently higher across almost all exposure categories, with the greatest risk in current smokers with heavy exposure (e.g., ≥20 cigarettes/day: HR 1.267; ≥20 pack-years: HR 1.220). Even former smokers with low exposure (<10 pack-years) showed increased risk (HR 1.093).

Overall, cumulative smoking exposure over time had a stronger influence on LSS risk than current smoking status alone. Notably, among adults aged ≥65 years, smoking cessation was associated with a meaningful reduction in risk, supporting the benefits of cessation even in late life.

**Figure A3:**
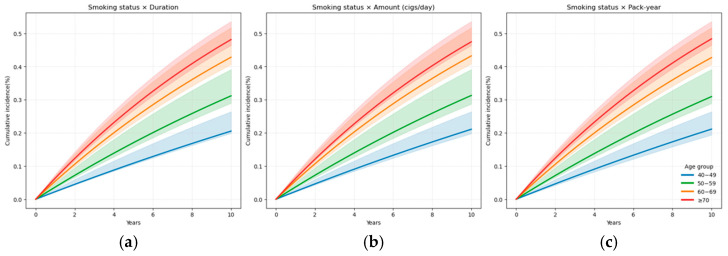
Cumulative incidence of LSS over a 10-year follow-up period by combined smoking status and exposure level across 10-year age bands. (**a**) Smoking duration; (**b**) Smoking amount (cigarettes/day); (**c**) Pack-year. Curves are stratified by age group (40–49, 50–59, 60–69, ≥70 years) and further divided into former (Ex) and current smokers. Non-smokers served as the reference group and are not displayed for visual clarity. Exposure subgroups are defined as <10, <20, and ≥20 years for smoking duration; <10, <20, and ≥20 cigarettes/day for smoking amount; and <10, <20, <30, <40, and ≥40 pack-years for pack-year Note: Numbers at risk at standard time points could not be displayed beneath the curves due to the use of aggregated summary-level data. Instead, group-specific participant numbers and events are provided in Table A5 of Appendix B, which can be cross-referenced for at-risk denominators.

**Figure A4:**
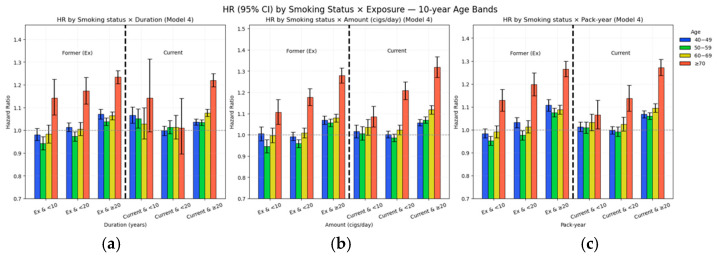
Hazard ratios (HRs) with 95% confidence intervals for LSS by combined smoking status and exposure level across 10-year age bands (Model 4). (**a**) Smoking duration; (**b**) Smoking amount (cigarettes/day); (**c**) Pack-year. Bars are grouped by age (40–49, 50–59, 60–69, ≥70 years) and arranged by exposure subgroup, with former (Ex) on the left and current on the right separated by a vertical dashed divider. The horizontal dashed line indicates HR = 1.0. Non-smokers served as the reference (HR = 1.0) and are not displayed for visual clarity. HRs were estimated from the fully adjusted Cox model (Model 4), adjusting for age, sex, body mass index, income level, alcohol consumption, regular exercise, diabetes mellitus, hypertension, and dyslipidemia.

Additional detailed stratified analyses using 10-year age bands (40–49, 50–59, 60–69, and ≥70 years) are provided in Table A5 and Figure A3 and Figure A4 of Appendix B. These analyses further highlight nuanced age-related variations, including elevated risk among former smokers in their 60s and the highest risk among current smokers with heavy exposure in the ≥70 age group.

#### 3.3.2. Sex-Stratified Effects of Combined Smoking Burden and Cessation

Sex-specific patterns were evident in the association between combined smoking burden and the risk of LSS. In men, prolonged smoking history was associated with a sustained elevation in risk, even after cessation. Former smokers with ≥20 years of smoking exhibited a hazard ratio (HR) of 1.080 (95% confidence interval [CI] 1.069–1.091), and current smokers with the same duration had an HR of 1.049 (95% CI 1.040–1.059). Interestingly, current smokers with 10–19 years of smoking demonstrated a reduced risk (HR 0.931, 95% CI 0.914–0.948). For heavy exposure, defined as ≥20 cigarettes/day or ≥20 pack-years, both former smokers (HR 1.084 and HR 1.109, respectively) and current smokers (HR 1.073 and HR 1.079, respectively) had significantly elevated risks. Across these high-exposure categories, former smokers consistently showed slightly higher risk than current smokers, suggesting the presence of irreversible damage and a possible “sick quitter effect.”

In women, current smoking emerged as a strong and independent risk factor across all categories of smoking duration, daily amount, and pack-years. Hazard ratios for current smokers ranged from 1.129 to 1.191 by duration, with the highest risks observed in those smoking ≥20 cigarettes/day (HR 1.266, 95% CI 1.217–1.316) and those with ≥20 pack-years (HR 1.195, 95% CI 1.142–1.251). In contrast, former smokers generally exhibited no significant excess risk, except for those with ≥20 cigarettes/day (HR 1.091, 95% CI 1.013–1.174) or 10–19 pack-years (HR 1.097, 95% CI 1.015–1.186), indicating a more immediate and pronounced risk reduction following cessation compared to men.

Overall, men demonstrated persistent risk following long-term heavy smoking despite cessation, whereas in women, the adverse effects were driven primarily by current smoking, with cessation associated with rapid and substantial attenuation of risk.

**Figure 12:**
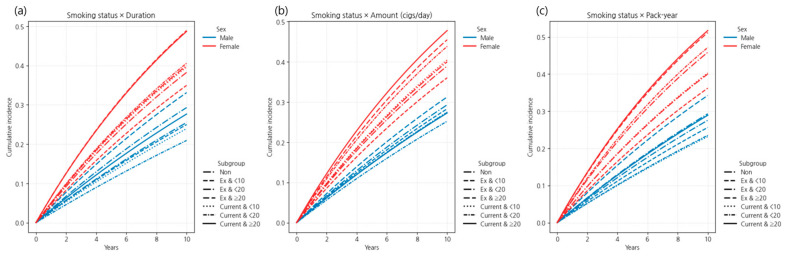
Cumulative incidence of LSS by combined smoking status and exposure level, stratified by sex: (**a**) smoking duration, (**b**) smoking amount (cigarettes/day), and (**c**) pack-years. Curves are based on a 10-year follow-up, with former smokers shown in blue and current smokers in red. Different line styles indicate exposure level categories within each smoking variable. Note: Numbers at risk at standard time points could not be displayed beneath the curves due to the use of aggregated summary-level data. Instead, group-specific participant numbers and events are provided in Table A6 of Appendix B, which can be cross-referenced for at-risk denominators.

**Figure 13:**
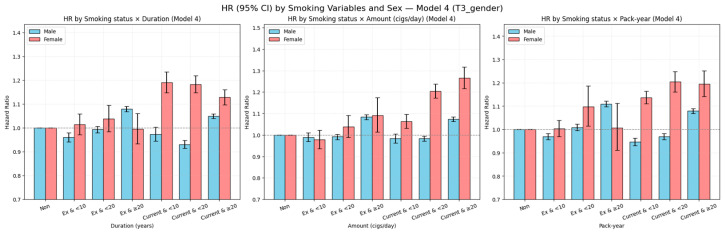
Hazard ratios (HRs) with 95% confidence intervals for LSS by combined smoking status and exposure level, stratified by sex (Model 4). Analyses were adjusted for age, body mass index, income, alcohol consumption, regular exercise, diabetes, hypertension, and dyslipidemia. The horizontal dashed line represents HR = 1.0; error bars indicate 95% confidence intervals.

These sex-specific trends were consistently observed in both cumulative incidence curves and adjusted hazard ratio plots (Figure 12 and Figure 13), with detailed numerical estimates provided in Table A6 of Appendix B. Sex-specific incidence rates revealed a consistent gap across all smoking exposure categories. Among current smokers with ≥20 pack-years, the rate was 72.8 per 1000 person-years in women versus 34.1 in men; for ≥20 cigarettes/day, 65.0 vs. 31.9; and for ≥20 years, 66.8 vs. 32.4. Even among former smokers with ≥20 pack-years, the gap persisted (71.7 in women vs. 41.9 in men). Non-smokers also showed a notable difference: 51.1 in women vs. 34.7 in men (Table A6 of Appendix B).

### 3.4. Association of Smoking with LSS Risk by Subgroups and Interaction Analysis

Lastly, subgroup and interaction analyses were conducted to examine whether the effect of smoking differed significantly according to major demographic and clinical characteristics. The results are presented in Table A7.

**Figure 14:**
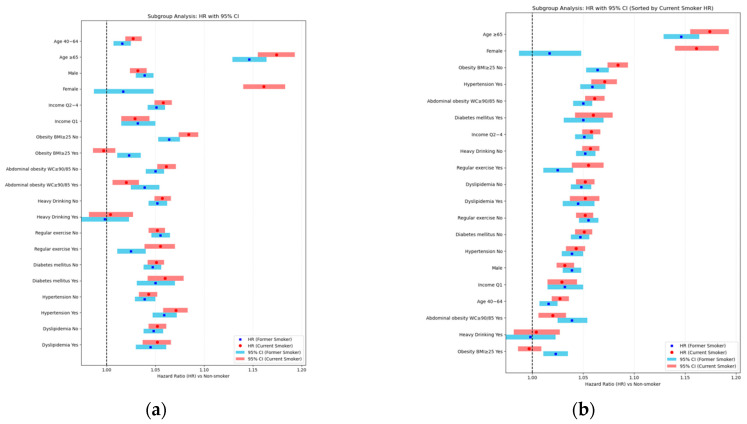
Multivariable subgroup analysis of the association between smoking and risk of LSS (Model 4). (**a**) Hazard ratios (HRs) with 95% confidence intervals (CIs) for former and current smokers compared with never smokers, presented by subgroup in the original variable order (Yes/No). This format allows direct comparison between categories within each variable. (**b**) Same data as in (**a**), but subgroups are sorted in descending order of HR for current smokers, highlighting groups with the greatest relative risk (e.g., age ≥65 years, female sex). HRs were derived from the fully adjusted Cox proportional hazards model (Model 4), adjusting for age, sex, body mass index, income level, alcohol consumption, regular exercise, diabetes mellitus, hypertension, and dyslipidemia. Horizontal bars represent 95% CIs, with red indicating current smokers and blue indicating former smokers.

Several demographic and clinical factors significantly modified the association between smoking and LSS risk. The strongest effect modification was observed for age and sex. Among participants aged ≥65 years, current and former smoking increased the risk by more than 14%, whereas in those aged 40–64 years, the increase was minimal. Women showed a markedly higher risk with current smoking (+16.1%) compared to men (+3.2%), while former smoking increased risk only in men. The interaction between sex and smoking status was statistically significant (*p* for interaction <0.0001), as shown in Appendix C.1, indicating that the association of smoking with LSS risk was significantly stronger in women than in men. Other significant modifiers included income, obesity status (by BMI and waist circumference), hypertension, heavy drinking, and regular exercise. In contrast, the associations were consistent across diabetes and dyslipidemia subgroups. Detailed subgroup-specific hazard ratios and interaction *p*-values are provided in Figure 14.

These results are visually summarized in Figure 14, which displays the hazard ratios and 95% confidence intervals for each subgroup in both the original variable order and in descending order of hazard ratio for current smokers, highlighting the groups with the greatest relative risk.

## 4. Discussion

For illustration, if the crude incidence rate in never smokers is approximately 46 per 1000 person-years, a 5% relative increase would correspond to 2–3 additional cases per 1000 person-years. Although modest at the individual level, this becomes a considerable burden at the population level given the high prevalence of smoking.

A clear dose–response pattern reinforces that cumulative lifetime smoking—more than current status alone—is the dominant driver of LSS risk, with the highest risk observed in heavy long-term smokers. Accordingly, counseling should quantify lifetime exposure (e.g., pack-years) and prioritize cessation for individuals with a substantial cumulative burden.

Despite extensive multivariable adjustment, residual confounding cannot be excluded, particularly from unmeasured factors such as prior spine trauma, occupational mechanical load (e.g., heavy lifting, vibration), analgesic use, menopausal/hormonal status, secondhand smoke exposure, and genetic predisposition. As an interpretive aid, the E-value for the main HR ≈ 1.05 is ~1.29, implying that an unmeasured confounder associated with both smoking and LSS by a risk ratio of ~1.3 each could, in principle, explain away the association.

The pathophysiology of lumbar spinal stenosis primarily involves the progressive narrowing of the spinal canal, resulting in the compression of neural elements [12]. This narrowing is predominantly driven by degenerative changes, including intervertebral disk degeneration, hypertrophy of the ligamentum flavum, and facet joint arthropathy, which collectively reduce canal diameter. Additional mechanisms, such as reduced microvascular perfusion from atherosclerotic changes, direct mechanical compression, and chronic inflammation, further exacerbate structural deterioration [13]. The observed higher prevalence of dyslipidemia and diabetes among current smokers likely reflects their long-term and heavy smoking history. These metabolic abnormalities promote atherosclerotic microvascular injury and reduced perfusion, providing a biologically plausible mechanism through which heavy or long-term smoking accelerates spinal degeneration and increases the risk of LSS [14,15].

These findings are also consistent with prior international cohort studies, reinforcing the biological plausibility of a smoking–LSS relationship across different populations. Rather than reflecting cultural or healthcare system differences, this consistency suggests a shared underlying pathophysiologic mechanism by which cumulative tobacco exposure accelerates spinal degeneration [8,9,12,16,17].

Tobacco smoking is a major preventable health hazard and is conclusively associated with multiple chronic disorders, including cancer, coronary artery disease, chronic obstructive pulmonary disease, and peripheral vascular disease [18]. In orthopedic and other surgical populations, smoking is well recognized as a risk factor for perioperative complications, such as wound infections, delayed healing, respiratory infections, and myocardial infarction [19]. In Korea, smoking accounted for more than 48,000 deaths in 2019, representing 22.7% of all fatalities that year, underscoring the substantial burden on the healthcare system [20].

After full multivariable adjustment, cigarette smoking remained an independent risk factor for LSS, indicating that the observed association is unlikely to be explained solely by demographic or lifestyle differences. This pattern underscores that the duration and intensity of smoking—rather than smoking status alone—drive spinal degeneration. In particular, long-term and heavy smoking appears to induce vascular and structural changes that may persist even after cessation, which helps explain the residual risk observed among former smokers.

The susceptibility to smoking-related risk was not uniform across the population. Older adults and women demonstrated a disproportionately higher vulnerability, suggesting an interaction between tobacco-related microvascular damage and age-related spinal degeneration, as well as potential hormonal or metabolic factors that may amplify the adverse effects of smoking. These findings highlight that the biological impact of smoking on the spine is modified by both age and sex, reinforcing the need for tailored preventive counseling in high-risk subgroups.

These findings extend prior international evidence by showing that the association between smoking and LSS persists after rigorous adjustment for lifestyle and metabolic factors. By leveraging multidimensional smoking metrics in a large, nationally representative cohort, our study demonstrates that long-term cumulative exposure—not smoking status alone—underlies the biological pathway linking smoking to spinal degeneration.

From a clinical standpoint, these findings underscore the importance of incorporating smoking history into LSS risk assessment and patient counseling, particularly for older adults and women, who constitute key high-risk subgroups. In orthopedic practice, preoperative screening for smoking status in patients scheduled for lumbar decompression or fusion surgery could help identify those at higher risk for postoperative complications and long-term functional decline. Furthermore, the observed dose–response relationship supports the potential benefit of smoking cessation programs not only for cardiovascular and pulmonary health but also for the prevention of degenerative spinal disorders.

Several limitations of this study warrant consideration. First, a key limitation is that smoking status and exposure were self-reported and assessed only at baseline (2009). With a median follow-up of over 10 years, we could not account for changes in smoking behavior, such as cessation, relapse, or changes in intensity, which may have led to misclassification bias. However, such nondifferential misclassification would likely have attenuated the true associations, suggesting that the reported risks may be underestimates. We also acknowledge that a “sick quitter effect” may exist, particularly among former smokers who show a higher risk than their current smoking counterparts. Second, although we implemented a one-year washout period to reduce reverse causation, residual bias cannot be completely excluded. Third, the Korean National Health Insurance System (NHIS) database, which is based on claims records, lacks detailed information on the severity of the disease, specific imaging findings (e.g., degree of canal narrowing, spinal alignment, or prior trauma history), or surgical intervention. Consequently, we could not differentiate between patients with mild versus severe stenosis or those requiring surgical treatment. However, our diagnostic criteria, which require a physician-assigned diagnosis and three or more claims, suggest that our outcome measure represents a clinically meaningful event rather than a mere radiographic finding. In addition, we acknowledge the potential for multicollinearity among smoking variables (duration, amount, pack-years); to minimize bias, these variables were not entered simultaneously into the same model but analyzed separately. Finally, as our cohort consisted exclusively of Korean adults, the generalizability of these findings beyond Korea may be limited. Differences in healthcare systems, smoking patterns, and demographic structures across countries should be considered when extrapolating these results. The NHIS data do not include menopausal status/timing, use of hormone therapy, or osteoporosis measurements; thus, these sex-specific pathways could not be quantified and causal interpretation warrants caution. Beyond acknowledging the absence of several potential confounders, the likely direction of residual bias is as follows. First, cigarette smoking is consistently associated with lower bone mineral density (BMD) and greater bone loss and fracture risk in a dose-dependent manner; lack of BMD adjustment may therefore have biased the smoking–LSS association upward (positive residual confounding) [21]. Second, heavy occupational mechanical loading (e.g., frequent heavy lifting, vibration) is linked to higher LSS risk, and such jobs tend to be more common among smokers; failure to measure occupational load likely overestimates the association [22]. Third, menopausal status and estrogen exposure influence lumbar disk degeneration, particularly within the first 15 years after menopause; unmeasured menopausal factors could amplify the association among women (positive confounding) or act as effect modifiers of the smoking–LSS relationship [23]. Finally, secondhand smoke has been associated with impaired bone health and higher fracture risk; if correlated with active smoking in our cohort, unmeasured environmental exposure could also bias estimates upward [24]. Taken together, some unmeasured factors likely bias estimates away from the null; however, the direction may vary by sex-specific pathways, and our sex-stratified analyses may mitigate but do not eliminate such bias.

In addition, because survival analyses were based on aggregated summary-level data, numbers at risk could not be shown beneath the cumulative incidence curves. Instead, group-specific participant counts and events were provided in the Appendix A Table A3 to support interpretability.

Despite these limitations, the strengths of our study include its large sample size, nationwide coverage, extended follow-up, comprehensive adjustment for demographic and clinical variables, and the ability to examine detailed exposure–response patterns using multiple smoking indicators.

Given the observed association between long-term smoking and the increased risk of lumbar spinal stenosis, our findings underscore the importance of smoking cessation or reduction as part of preventive strategies for degenerative spinal disorders. Counseling patients—particularly older adults—on smoking cessation may contribute not only to general cardiovascular and metabolic health, but also to reducing the burden of spinal stenosis in the aging population. This reinforces that smoking cessation should be emphasized not only for cardiovascular and metabolic outcomes, but also as part of musculoskeletal health preservation strategies.

In addition to influencing disease onset, smoking also has important implications for postoperative recovery and structural healing within the spine. Cigarette smoking is well known to impair bone healing and delay spinal fusion, meaning that it can hinder not only prevention efforts but also postsurgical outcomes. Accordingly, counseling strategies should emphasize both the prevention of disease progression and optimization of postoperative recovery. Educating patients about the detrimental effects of smoking on bone regeneration and supporting cessation efforts may improve fusion success and overall postoperative outcomes.

In conclusion, our study provides robust epidemiologic evidence that cigarette smoking was independently associated with LSS, reinforcing its role as a modifiable risk factor. The magnitude of risk is particularly high among older adults, women, and long-term heavy smokers. These findings highlight the potential role of smoking cessation strategies in mitigating spinal degeneration and improving quality of life in at-risk populations. Future prospective studies incorporating imaging biomarkers, longitudinal exposure assessment, and biochemical measures of disk degeneration will be essential to more precisely delineate the biological pathways through which smoking contributes to lumbar spinal degeneration.

## Figures and Tables

**Table 1:** Baseline characteristics of study participants according to smoking status.

	Never Smoker	Former Smoker	Current Smoker	*p*-Value
n	1,306,249	339,915	477,104	
**Age groups**				<0.0001
40–64	1,099,107 (84.14)	289,693 (85.23)	432,328 (90.62)	
≥65	207,142 (15.86)	50,222 (14.77)	44,776 (9.38)	
**Sex**				<0.0001
Male	357,217 (27.35)	328,631 (96.68)	449,310 (94.17)	
Female	949,032 (72.65)	11,284 (3.32)	27,794 (5.83)	
**Income, Lowest Q1**	293,799 (22.49)	49,967 (14.7)	87,388 (18.32)	<0.0001
**BMI Level**				<0.0001
<18.5	31,271 (2.39)	4627 (1.36)	14,090 (2.95)	
<23	517,639 (39.63)	97,415 (28.66)	176,102 (36.91)	
<25	339,453 (25.99)	99,144 (29.17)	125,576 (26.32)	
<30	375,050 (28.71)	128,663 (37.85)	148,090 (31.04)	
≥30	42,836 (3.28)	10,066 (2.96)	13,246 (2.78)	
**Alcohol consumption**				<0.0001
None	954,210 (73.05)	106,525 (31.34)	122,164 (25.61)	
Moderate	319,827 (24.48)	187,678 (55.21)	266,570 (55.87)	
Heavy	32,212 (2.47)	45,712 (13.45)	88,370 (18.52)	
**Regular exercise**	249,562 (19.11)	92,889 (27.33)	86,231 (18.07)	<0.0001
**Diabetes mellitus**	123,399 (9.45)	46,555 (13.7)	62,482 (13.1)	<0.0001
**Hypertension**	390,577 (29.9)	124,983 (36.77)	140,539 (29.46)	<0.0001
**Dyslipidemia**	276,664 (21.18)	76,057 (22.38)	94,100 (19.72)	<0.0001
**Age, years**	53.38 ± 10.16	53.42 ± 9.72	50.89 ± 8.95	<0.0001
**Height, cm**	158.88 ± 7.89	168.24 ± 6.23	168 ± 6.63	<0.0001
**Weight, kg**	60.16 ± 9.85	69.19 ± 9.62	67.36 ± 10.4	<0.0001
**BMI, kg/m^2^**	23.77 ± 3.06	24.4 ± 2.8	23.81 ± 3.03	<0.0001
**Waist Circumference, cm**	79.14 ± 8.64	84.69 ± 7.43	83.29 ± 7.86	<0.0001
**Systolic BP, mmHg**	122.9 ± 15.74	126.19 ± 14.58	124.67 ± 15.05	<0.0001
**Diastolic BP, mmHg**	76.25 ± 10.3	78.92 ± 9.93	78.29 ± 10.23	<0.0001
**Fasting glucose, mg/dL**	98.18 ± 23.76	102.87 ± 27.04	102.52 ± 30.45	<0.0001
**Total Cholesterol, mg/dL**	198.81 ± 36.93	197.91 ± 36.81	198.61 ± 37.38	<0.0001
**HDL-C, mg/dL**	57.28 ± 30.43	53.42 ± 25.44	52.98 ± 26.26	<0.0001
**LDL-C, mg/dL**	118.27 ± 38.32	115.1 ± 37.79	112.97 ± 39.36	<0.0001
**Triglyceride, mg/dL**	106.45 (106.35–106.55)	129.56 (129.32–129.81)	142.5 (142.27–142.73)	<0.0001

Data are presented as mean ± standard deviation or n (%). Data are presented as median (interquartile range). *p*-values were derived from one-way analysis of variance (ANOVA) for continuous variables and the chi-square test for categorical variables. Abbreviations: BMI, body mass index; HDL-C, high-density lipoprotein cholesterol; LDL-C, low-density lipoprotein cholesterol.

**Table 2:** Association between smoking-related exposure and risk of LSS.

	N	Event	Duration	IR, per 1000 PY	HR (95% C.I)			
					Model 1	Model 2	Model 3	Model 4
**Smoking**								
None	1,306,249	488,735	10,527,390.24	46.4251	1 (Ref.)	1 (Ref.)	1 (Ref.)	1 (Ref.)
Former	339,915	101,758	2,877,221.61	35.3668	0.761 (0.756, 0.766)	1.064 (1.056, 1.073)	1.049 (1.040, 1.058)	1.047 (1.039, 1.056)
Current	477,104	131,416	4,077,436.53	32.2301	0.694 (0.689, 0.698)	1.054 (1.046, 1.062)	1.054 (1.045, 1.062)	1.052 (1.044, 1.060)
*p*-value					<0.0001	<0.0001	<0.0001	<0.0001
**Smoking duration**								
None	1,306,249	488,735	10,527,390.24	46.4251	1 (Ref.)	1 (Ref.)	1 (Ref.)	1 (Ref.)
<10	75,749	20,695	660,591.29	31.328	0.674 (0.665, 0.684)	1.008 (0.994, 1.023)	1.005 (0.991, 1.020)	1.005 (0.990, 1.019)
<20	182,604	46,539	1,614,862.4	28.8192	0.620 (0.615, 0.626)	1.013 (1.002, 1.024)	1.003 (0.992, 1.014)	1.001 (0.991, 1.012)
≥20	558,666	165,940	4,679,204.45	35.4633	0.763 (0.759, 0.768)	1.082 (1.074, 1.090)	1.076 (1.068, 1.084)	1.074 (1.066, 1.082)
*p*-value					<0.0001	<0.0001	<0.0001	<0.0001
**Smoking amount**								
None	1,306,249	488,735	10,527,390.24	46.4251	1 (Ref.)	1 (Ref.)	1 (Ref.)	1 (Ref.)
<10	87,076	25,932	727,072.87	35.6663	0.768 (0.758, 0.777)	0.999 (0.986, 1.012)	1.007 (0.994, 1.020)	1.007 (0.994, 1.020)
<20	301,014	81,915	2,595,251.76	31.5634	0.679 (0.674, 0.684)	1.009 (1.000, 1.018)	1.013 (1.004, 1.022)	1.011 (1.003, 1.020)
≥20	428,929	125,327	3,632,333.51	34.5032	0.743 (0.738, 0.747)	1.118 (1.109, 1.127)	1.097 (1.088, 1.106)	1.095 (1.086, 1.104)
*p*-value					<0.0001	<0.0001	<0.0001	<0.0001
**Pack-year**								
None	1,306,249	488,735	10,527,390.24	46.4251	1 (Ref.)	1 (Ref.)	1 (Ref.)	1 (Ref.)
<10	194,823	52,573	1,695,866.69	31.0007	0.667 (0.661, 0.673)	0.996 (0.986, 1.006)	0.996 (0.986, 1.006)	0.995 (0.985, 1.005)
<20	231,190	60,742	2,015,666.62	30.1349	0.649 (0.643, 0.654)	1.016 (1.006, 1.026)	1.013 (1.003, 1.023)	1.011 (1.001, 1.021)
<30	194,542	52,777	1,677,908.99	31.454	0.677 (0.671, 0.683)	1.057 (1.046, 1.068)	1.047 (1.037, 1.058)	1.045 (1.034, 1.056)
<40	112,150	35,842	924,688.79	38.7611	0.835 (0.826, 0.844)	1.141 (1.128, 1.155)	1.127 (1.114, 1.141)	1.125 (1.112, 1.139)
≥40	84,314	31,240	640,527.06	48.7723	1.052 (1.040, 1.064)	1.223 (1.208, 1.238)	1.208 (1.193, 1.224)	1.207 (1.191, 1.222)
*p*-value					<0.0001	<0.0001	<0.0001	<0.0001

IR, incidence rate (per 1000 person-years); HR, hazard ratio; CI, confidence interval. Model 1: unadjusted; Model 2: adjusted for age and sex; Model 3: Model 2 + income, body mass index (BMI), alcohol consumption, and regular physical activity; Model 4: Model 3 + diabetes mellitus, hypertension, and dyslipidemia. Incidence rates are crude (unadjusted), presented per 1000 person-years. Incident LSS was defined as ≥3 M48.06 claims on distinct service dates (outpatient or inpatient; same-day duplicates counted once; same provider not required); index date = date of the third claim.

**Table 3:** Hazard ratios (HRs) and 95% confidence intervals (CIs) for LSS by combined smoking status and cumulative smoking exposure: duration, amount, and pack-year.

	N	Event	Duration	IR, per 1000	HR (95% C.I)			
					Model 1	Model 2	Model 3	Model 4
**Smoking status and Duration**								
None	1,306,249	488,735	10,527,390.24	46.4251	1 (Ref.)	1 (Ref.)	1 (Ref.)	1 (Ref.)
Ex and <10	50,731	13,488	445,496.19	30.2764	0.652 (0.641, 0.663)	0.981 (0.964, 0.998)	0.979 (0.962, 0.996)	0.978 (0.961, 0.995)
Ex and <20	110,117	29,059	969,121.1	29.9849	0.645 (0.638, 0.653)	1.024 (1.011, 1.037)	1.008 (0.995, 1.021)	1.007 (0.994, 1.020)
Ex and ≥20	179,067	59,211	1,462,604.32	40.4833	0.872 (0.865, 0.879)	1.112 (1.101, 1.123)	1.093 (1.082, 1.104)	1.091 (1.080, 1.102)
Current and <10	25,018	7207	215,095.1	33.5061	0.721 (0.704, 0.738)	1.063 (1.038, 1.088)	1.058 (1.033, 1.083)	1.057 (1.032, 1.082)
Current and <20	72,487	17,480	645,741.3	27.0697	0.583 (0.574, 0.592)	0.994 (0.979, 1.010)	0.992 (0.977, 1.008)	0.991 (0.975, 1.006)
Current and ≥20	379,599	106,729	3,216,600.13	33.1807	0.714 (0.709, 0.719)	1.066 (1.057, 1.075)	1.066 (1.057, 1.075)	1.065 (1.056, 1.074)
**Smoking status and amount**								
None	1,306,249	488,735	10,527,390.24	46.4251	1 (Ref.)	1 (Ref.)	1 (Ref.)	1 (Ref.)
Ex and <10	40,665	12,027	345,340.08	34.8265	0.750 (0.736, 0.763)	0.993 (0.975, 1.011)	0.998 (0.979, 1.016)	0.997 (0.979, 1.016)
Ex and <20	128,117	35,973	1,105,256.62	32.5472	0.700 (0.693, 0.708)	1.011 (0.999, 1.023)	1.007 (0.995, 1.019)	1.006 (0.994, 1.017)
Ex and ≥20	171,133	53,758	1,426,624.91	37.6819	0.811 (0.804, 0.819)	1.132 (1.120, 1.143)	1.100 (1.089, 1.111)	1.097 (1.086, 1.109)
Current and <10	46,411	13,905	381,732.79	36.426	0.784 (0.771, 0.798)	1.004 (0.987, 1.021)	1.015 (0.998, 1.033)	1.015 (0.997, 1.032)
Current and <20	172,897	45,942	1,489,995.14	30.8337	0.664 (0.657, 0.670)	1.007 (0.996, 1.018)	1.017 (1.006, 1.028)	1.016 (1.005, 1.027)
Current and ≥20	257,796	71,569	2,205,708.61	32.4472	0.698 (0.693, 0.704)	1.107 (1.097, 1.118)	1.095 (1.084, 1.105)	1.092 (1.082, 1.103)
**Smoking status and Pack-year**								
None	1,306,249	488,735	10,527,390.24	46.4251	1 (Ref.)	1 (Ref.)	1 (Ref.)	1 (Ref.)
Ex and <10	112,688	30,325	985,606.07	30.7679	0.662 (0.655, 0.670)	0.988 (0.976, 1.000)	0.987 (0.974, 0.999)	0.986 (0.973, 0.998)
Ex and <20	99,797	28,095	861,820.86	32.5996	0.702 (0.693, 0.710)	1.043 (1.029, 1.056)	1.027 (1.014, 1.041)	1.025 (1.012, 1.039)
Ex and ≥20	127,430	43,338	1,029,794.68	42.0841	0.907 (0.898, 0.916)	1.155 (1.142, 1.168)	1.126 (1.113, 1.139)	1.123 (1.111, 1.136)
Current and <10	82,135	22,248	710,260.62	31.3237	0.674 (0.665, 0.683)	1.008 (0.995, 1.022)	1.011 (0.997, 1.026)	1.010 (0.996, 1.025)
Current and <20	131,393	32,647	1,153,845.75	28.2941	0.609 (0.603, 0.616)	0.995 (0.983, 1.007)	1.002 (0.990, 1.014)	1.000 (0.988, 1.013)
Current and ≥20	263,576	76,521	2,213,330.16	34.5728	0.744 (0.738, 0.750)	1.103 (1.093, 1.113)	1.098 (1.088, 1.109)	1.097 (1.087, 1.107)

Hazard ratios (HRs) and 95% confidence intervals (CIs) were estimated using the fully adjusted model (Model 4). Definitions of IR, HR, CI, Model 4, and reference group are provided in the footnote of Table 2. Smoking duration categories: <10, <20, ≥20 years; smoking amount: <10, <20, ≥20 cigarettes/day; pack-year: <10, <20, ≥20 pack-years. Incidence rates are crude (unadjusted), presented per 1000 person-years. Incident LSS was defined as ≥3 M48.06 claims on distinct service dates (outpatient or inpatient; same-day duplicates counted once; same provider not required); index date = date of the third claim.

## Data Availability

All data concerning the case are presented in the manuscript.

## Appendix A

### Appendix A.1. Association Between Smoking and LSS by Dichotomized Age Groups (<65 vs. ≥65 Years)

This appendix section provides the detailed numerical estimates for the primary age-stratified analysis described in Section 3.2.1. In the dichotomized analysis, both former and current smoking were associated with modest risk increases in younger adults (<65 years; HR 1.016 and 1.027, respectively), whereas in participants aged ≥ 65 years, the increases were more pronounced (HR 1.146 and 1.174, respectively). Results for smoking duration, daily amount, and pack-years showed a similar pattern, with stronger associations in older adults. The corresponding figures depicting cumulative incidence and adjusted hazard ratios are presented in the main text (Figure 4 and Figure 5).

**Table A1:** Association between smoking-related variables and risk of LSS by dichotomized age groups (<65 vs. ≥65 years).

	Age 40–64 Years					Age ≥65 Years				
	N	Event	Duration	IR, per 1000 PY	Model 4	N	Event	Duration	IR, per 1000 PY	Model 4
**Smoking**										
None	1,099,107	384,974	9,190,028.6	41.8904	1 (Ref.)	207,142	103,761	1,337,361.64	77.5863	1 (Ref.)
Former	289,693	80,465	2,537,191.47	31.7142	1.016 (1.007, 1.025)	50,222	21,293	340,030.13	62.6209	1.146 (1.129, 1.164)
Current	432,328	113,120	3,787,142.02	29.8695	1.027 (1.019, 1.036)	44,776	18,296	290,294.51	63.0256	1.174 (1.155, 1.193)
**Smoking duration**										
None	1,099,107	384,974	9,190,028.6	41.8904	1 (Ref.)	207,142	103,761	1,337,361.64	77.5863	1 (Ref.)
<10	70,412	18,446	623,498.34	29.5847	0.991 (0.976, 1.006)	5337	2249	37,092.96	60.6315	1.088 (1.043, 1.135)
<20	172,790	42,370	1,546,564.87	27.3962	0.988 (0.977, 0.999)	9814	4169	68,297.53	61.0417	1.115 (1.080, 1.150)
≥20	478,819	132,769	4,154,270.29	31.9596	1.043 (1.034, 1.051)	79,847	33,171	524,934.15	63.1908	1.172 (1.157, 1.188)
**Smoking amount**										
None	1,099,107	384,974	9,190,028.6	41.8904	1 (Ref.)	207,142	103,761	1,337,361.64	77.5863	1 (Ref.)
<10	71,438	19,640	624,696.7	31.4393	0.995 (0.980, 1.010)	15,638	6292	102,376.17	61.4596	1.057 (1.030, 1.084)
<20	266,882	67,873	2,365,595.98	28.6917	0.983 (0.974, 0.993)	34,132	14,042	229,655.78	61.1437	1.134 (1.114, 1.155)
≥20	383,701	106,072	3,334,040.82	31.8148	1.063 (1.054, 1.072)	45,228	19,255	298,292.7	64.5507	1.224 (1.204, 1.244)
**Pack-year**										
None	1,099,107	384,974	9,190,028.6	41.8904	1 (Ref.)	207,142	103,761	1,337,361.64	77.5863	1 (Ref.)
<10	178,284	45,617	1,583,458.76	28.8085	0.980 (0.969, 0.990)	16,539	6956	112,407.93	61.8818	1.085 (1.059, 1.112)
<20	211,197	52,527	1,880,898.38	27.9265	0.992 (0.982, 1.002)	19,993	8215	134,768.24	60.9565	1.117 (1.091, 1.142)
<30	175,174	44,869	1,548,869.83	28.9689	1.025 (1.014, 1.037)	19,368	7908	129,039.16	61.2837	1.150 (1.123, 1.177)
<40	98,322	30,092	832,115.32	36.1633	1.093 (1.079, 1.107)	13,828	5750	92,573.46	62.1128	1.176 (1.144, 1.208)
≥40	59,044	20,480	478,991.21	42.7565	1.170 (1.152, 1.188)	25,270	10,760	161,535.85	66.6106	1.265 (1.239, 1.291)

Hazard ratios (HRs) and 95% confidence intervals (CIs) were estimated using the fully adjusted model (Model 4). IR and variable definitions are as described in the footnote of Table 2. Incidence rates are crude (unadjusted), presented per 1000 person-years. Incident LSS was defined as ≥3 M48.06 claims on distinct service dates (outpatient or inpatient; same-day duplicates counted once; same provider not required); index date = date of the third claim.

### Appendix A.2. Association Between Smoking and LSS by 10-Year Age Bands (40–49, 50–59, 60–69, and ≥70 Years)

Stratification by 10-year age bands revealed progressive increases in smoking-related risk with advancing age. In adults aged 40–49 years, only small but statistically significant elevations were observed for both former (HR 1.026; 95% CI 1.012–1.040) and current smoking (HR 1.029; 95% CI 1.017–1.040). Among those aged 60–69 and ≥70 years, smoking-related risk became more pronounced, with hazard ratios exceeding 1.20 for heavy or prolonged exposure. Detailed estimates are tabulated below, with cumulative incidence curves and adjusted hazard ratios summarized graphically

**Table A2:** Association between smoking-related variables and risk of LSS by 10-year age groups.

Age Group	Category	N	Events	Duration (PY)	IR (per 1000 PY)	Model 4 HR (95% CI)
**40–49 years**	**Smoking**					
	None	544,195	148,119	4,834,353.75	30.64	1 (Ref.)
	Former	137,294	29,398	1,261,274.29	23.31	1.026 (1.012–1.040)
	Current	246,597	51,943	2,254,426.63	23.04	1.029 (1.017–1.040)
	**Smoking Duration**					
	<10	43,090	9430	393,815.87	23.95	1.013 (0.992–1.035)
	<20	115,116	23,654	1,059,616.41	22.32	1.007 (0.992–1.022)
	≥20	225,685	48,257	2,062,268.65	23.4	1.045 (1.033–1.058)
	**Smoking Amount**					
	<10	38,649	8536	352,502.35	24.22	1.011 (0.989–1.034)
	<20	147,657	30,050	1,361,334.10	22.07	0.998 (0.985–1.012)
	≥20	197,585	42,755	1,801,864.46	23.73	1.061 (1.049–1.074)
	**Pack-year**					
	<10	108,482	22,804	996,145.73	22.89	0.999 (0.984–1.013)
	<20	126,740	25,766	1,168,249.29	22.06	1.012 (0.997–1.026)
	<30	105,835	22,383	968,373.34	23.11	1.046 (1.031–1.062)
	<40	29,360	6968	263,178.05	26.48	1.137 (1.109–1.166)
	≥40	13,474	3420	119,754.50	28.56	1.198 (1.158–1.240)
**5** **0–** **5** **9 years**	**Smoking**					
	None	411,790	164,725	3,317,386.08	49.66	1 (Ref.)
	Former	112,843	34,982	967,675.15	36.15	1.005 (0.993–1.018)
	Current	146,289	45,375	1,233,935.28	36.77	1.032 (1.020–1.044)
	**Smoking Duration**					
	<10	22,069	6894	188,610.06	36.55	0.980 (0.956–1.004)
	<20	46,915	14,410	402,247.15	35.82	0.987 (0.970–1.005)
	≥20	190,148	59,053	1,610,753.22	36.66	1.036 (1.025–1.048)
	**Smoking Amount**					
	<10	24,127	7588	205,330.79	36.96	0.976 (0.954–0.999)
	<20	92,067	27,270	791,947.52	34.43	0.974 (0.961–0.988)
	≥20	142,938	45,499	1,204,332.13	37.78	1.065 (1.052–1.077)
	**Pack-year**					
	<10	55,429	17,074	474,632.40	35.97	0.975 (0.959–0.991)
	<20	67,291	19,986	578,502.12	34.55	0.985 (0.970–1.001)
	<30	54,810	16,669	468,546.87	35.58	1.020 (1.003–1.037)
	<40	54,274	17,254	456,174.14	37.82	1.075 (1.057–1.093)
	≥40	27,328	9374	223,754.91	41.89	1.146 (1.121–1.171)
**6** **0–** **6** **9 years**	**Smoking**					
	None	234,680	120,838	1,658,865.29	72.84	1 (Ref.)
	Former	63,700	26,516	483,507.74	54.84	1.043 (1.028–1.057)
	Current	62,576	25,584	459,445.93	55.68	1.070 (1.055–1.085)
	**Smoking Duration**					
	<10	8086	3325	62,011.07	53.62	0.995 (0.961–1.030)
	<20	16,114	6629	123,900.78	53.5	1.008 (0.983–1.034)
	≥20	102,076	42,146	757,041.83	55.67	1.071 (1.059–1.085)
	**Smoking Amount**					
	<10	15,304	6349	114,615.22	55.39	1.016 (0.990–1.042)
	<20	43,653	17,490	330,843.13	52.86	1.017 (1.000–1.034)
	≥20	67,319	28,261	497,495.32	56.81	1.097 (1.082–1.113)
	**Pack-year**					
	<10	22,502	9291	171,676.93	54.12	1.006 (0.984–1.027)
	<20	27,136	10,957	206,175.28	53.14	1.018 (0.998–1.039)
	<30	23,692	9655	177,381.99	54.43	1.053 (1.031–1.076)
	<40	22,014	8976	165,051.01	54.38	1.064 (1.041–1.087)
	≥40	30,932	13,221	222,668.46	59.38	1.146 (1.125–1.168)
**≥70 years**	**Smoking**					
	None	115,584	55,053	716,785.13	76.81	1 (Ref.)
	Former	26,078	10,862	164,764.43	65.92	1.215 (1.190–1.241)
	Current	21,642	8514	129,628.69	65.68	1.209 (1.181–1.237)
	**Smoking Duration**					
	<10	2504	1046	16,154.30	64.75	1.144 (1.076–1.216)
	<20	4459	1846	29,098.07	63.44	1.147 (1.095–1.202)
	≥20	40,757	16,484	249,140.75	66.16	1.227 (1.206–1.250)
	**Smoking Amount**					
	<10	8996	3459	54,624.50	63.32	1.094 (1.057–1.133)
	<20	17,637	7105	111,127.01	63.94	1.192 (1.163–1.223)
	≥20	21,087	8812	128,641.61	68.5	1.293 (1.263–1.323)
	**Pack-year**					
	<10	8410	3404	53,411.62	63.73	1.106 (1.068–1.146)
	<20	10,023	4033	62,739.92	64.28	1.172 (1.135–1.211)
	<30	10,205	4070	63,606.79	63.99	1.212 (1.174–1.252)
	<40	6502	2644	40,285.59	65.63	1.232 (1.185–1.281)
	≥40	12,580	5225	74,349.19	70.28	1.339 (1.301–1.378)

Hazard ratios (HRs) and 95% confidence intervals (CIs) were estimated using the fully adjusted model (Model 4), adjusting for age, sex, body mass index, income level, alcohol consumption, regular exercise, diabetes mellitus, hypertension, and dyslipidemia. IR: incidence rate per 1000 person-years. Incidence rates are crude (unadjusted), presented per 1000 person-years. Incident LSS was defined as ≥3 M48.06 claims on distinct service dates (outpatient or inpatient; same-day duplicates counted once; same provider not required); index date = date of the third claim.

### Appendix A.3. Detailed Hazard Ratios for LSS by Smoking Exposure Categories, Stratified by Sex

Detailed numerical results of the sex-stratified analysis described in Section 3.2.2 are presented below.

**Table A3:** Hazard ratios (HRs) and 95% confidence intervals (CIs) for LSS by smoking-related variables, stratified by sex (Model 4).

	Male					Female				
	N	Event	Duration	IR, per 1000 PY	Model 4	N	Event	Duration	IR, per 1000 PY	Model 4
**Smoking**										
None	357,217	104,334	3,007,774.09	34.6881	1 (Ref.)	949,032	384,401	7,519,616.15	51.1198	1 (Ref.)
Former	328,631	97,404	2,787,349.58	34.945	1.039 (1.030, 1.048)	11,284	4354	89,872.02	48.4467	1.017 (0.987, 1.048)
Current	449,310	119,361	3,865,165.72	30.8812	1.032 (1.024, 1.041)	27,794	12,055	212,270.81	56.7907	1.161 (1.140, 1.183)
**Smoking duration**										
None	357,217	104,334	3,007,774.09	34.6881	1 (Ref.)	949,032	384,401	7,519,616.15	51.1198	1 (Ref.)
<10	62,808	15,751	555,552.11	28.352	0.964 (0.948, 0.981)	12,941	4944	105,039.19	47.0681	1.111 (1.080, 1.143)
<20	168,914	40,989	1,506,248.7	27.2126	0.973 (0.962, 0.984)	13,690	5550	108,613.7	51.0985	1.144 (1.114, 1.175)
≥20	546,219	160,025	4,590,714.5	34.8584	1.061 (1.052, 1.069)	12,447	5915	88,489.95	66.8438	1.105 (1.076, 1.133)
**Smoking amount**										
None	357,217	104,334	3,007,774.09	34.6881	1 (Ref.)	949,032	384,401	7,519,616.15	51.1198	1 (Ref.)
<10	70,864	19,610	598,874.88	32.7447	0.986 (0.971, 1.002)	16,212	6322	128,197.99	49.3143	1.034 (1.009, 1.060)
<20	284,975	75,060	2,471,802.95	30.3665	0.987 (0.978, 0.997)	16,039	6855	123,448.81	55.5291	1.161 (1.134, 1.189)
≥20	422,102	122,095	3,581,837.47	34.0873	1.078 (1.069, 1.087)	6827	3232	50,496.04	64.005	1.223 (1.181, 1.266)
**Pack-year**										
None	357,217	104,334	3,007,774.09	34.6881	1 (Ref.)	949,032	384,401	7,519,616.15	51.1198	1 (Ref.)
<10	167,949	42,010	1,481,513.03	28.3561	0.960 (0.949, 0.971)	26,874	10,563	214,353.66	49.2784	1.091 (1.070, 1.112)
<20	223,442	57,129	1,958,628.77	29.1679	0.987 (0.977, 0.998)	7748	3613	57,037.84	63.3439	1.184 (1.146, 1.224)
<30	191,803	51,456	1,658,332.31	31.0288	1.027 (1.016, 1.038)	2739	1321	19,576.68	67.4782	1.169 (1.107, 1.234)
<40	111,053	35,248	917,426.98	38.4205	1.108 (1.094, 1.121)	1097	594	7261.81	81.7978	1.222 (1.128, 1.325)
≥40	83,694	30,922	636,614.21	48.5726	1.192 (1.177, 1.208)	620	318	3912.85	81.2708	1.030 (0.923, 1.150)

Hazard ratios (HRs) and 95% confidence intervals (CIs) were estimated using the fully adjusted Cox proportional hazards model (Model 4). Definitions of IR, HR, CI, Model 4, and smoking categories are provided in the footnote of Table 2. Incidence rates are crude (unadjusted), presented per 1000 person-years. Incident LSS was defined as ≥3 M48.06 claims on distinct service dates (outpatient or inpatient; same-day duplicates counted once; same provider not required); index date = date of the third claim.

## Appendix B

### Appendix B.1. Age-Stratified Analysis of Combined Smoking Status and Cumulative Exposure (40–64 vs. ≥65 Years)

This section presents detailed age-stratified results for the combined effect of smoking status (never, former, current) and cumulative exposure (duration, daily amount, pack-years). Hazard ratios (HRs) and incidence rates (IRs) were estimated separately for two age groups (40–64 and ≥65 years), allowing a clearer comparison of risk profiles between middle-aged and older adults. These results supplement the summary provided in Section 3.3.1 and highlight age-related heterogeneity in the association between smoking burden and LSS.

**Table A4:** Hazard ratios (HRs) and 95% confidence intervals (CIs) for LSS by combined smoking status and cumulative exposure, stratified by age groups 40–64 and ≥65 years.

	Age 40–64 Years					Age ≥ 65 Years				
	N	Event	Duration	IR, per 1000 PY	Model 4	N	Event	Duration	IR, per 1000 PY	Model 4
**Smoking status and Duration**										
None	1,099,107	384,974	9,190,028.6	41.8904	1 (Ref.)	207,142	103,761	1,337,361.64	77.5863	1 (Ref.)
Ex and <10	46,560	11,739	416,092.6	28.2125	0.960 (0.942, 0.978)	4171	1749	29,403.59	59.4825	1.078 (1.028, 1.130)
Ex and <20	102,062	25,643	912,671.29	28.0966	0.987 (0.973, 1.000)	8055	3416	56,449.81	60.5139	1.121 (1.083, 1.160)
Ex and ≥20	141,071	43,083	1,208,427.59	35.6521	1.056 (1.044, 1.068)	37,996	16,128	254,176.73	63.4519	1.162 (1.142, 1.182)
Current and <10	23,852	6707	207,405.74	32.3376	1.048 (1.023, 1.074)	1166	500	7689.36	65.0249	1.122 (1.028, 1.226)
Current and <20	70,728	16,727	633,893.58	26.3877	0.988 (0.972, 1.004)	1759	753	11,847.72	63.5565	1.083 (1.008, 1.164)
Current and ≥20	337,748	89,686	2,945,842.71	30.4449	1.036 (1.027, 1.045)	41,851	17,043	270,757.42	62.9456	1.181 (1.162, 1.201)
**Smoking status and Amount**										
None	1,099,107	384,974	9,190,028.6	41.8904	1 (Ref.)	207,142	103,761	1,337,361.64	77.5863	1 (Ref.)
Ex and <10	33,869	9208	298,378.64	30.8601	0.976 (0.955, 0.996)	6796	2819	46,961.44	60.028	1.069 (1.030, 1.110)
Ex and <20	111,522	29,025	990,516.61	29.3029	0.976 (0.963, 0.988)	16,595	6948	114,740	60.5543	1.111 (1.084, 1.139)
Ex and ≥20	144,302	42,232	1,248,296.22	33.8317	1.063 (1.051, 1.075)	26,831	11,526	178,328.69	64.6335	1.196 (1.172, 1.220)
Current and <10	37,569	10,432	326,318.06	31.9688	1.012 (0.992, 1.032)	8842	3473	55,414.73	62.6729	1.046 (1.011, 1.082)
Current and <20	155,360	38,848	1,375,079.36	28.2515	0.989 (0.977, 1.000)	17,537	7094	114,915.78	61.7322	1.158 (1.130, 1.186)
Current and ≥20	239,399	63,840	2,085,744.6	30.6078	1.063 (1.052, 1.073)	18,397	7729	119,964.01	64.4277	1.267 (1.238, 1.298)
**Smoking status and Pack-year**										
None	1,099,107	384,974	9,190,028.6	41.8904	1 (Ref.)	207,142	103,761	1,337,361.64	77.5863	1 (Ref.)
Ex and <10	101,770	25,723	909,312.8	28.2884	0.963 (0.950, 0.976)	10,918	4602	76,293.27	60.3199	1.093 (1.061, 1.126)
Ex and <20	88,423	23,332	782,979.37	29.799	1.000 (0.986, 1.014)	11,374	4763	78,841.49	60.4124	1.118 (1.086, 1.151)
Ex and ≥20	99,500	31,410	844,899.3	37.176	1.091 (1.077, 1.105)	27,930	11,928	184,895.37	64.5122	1.187 (1.164, 1.210)
Current and <10	76,514	19,894	674,145.96	29.5099	1.003 (0.988, 1.017)	5621	2354	36,114.67	65.1813	1.070 (1.027, 1.114)
Current and <20	122,774	29,195	1,097,919.01	26.5912	0.985 (0.973, 0.998)	8619	3452	55,926.74	61.7236	1.114 (1.077, 1.153)
Current and ≥20	233,040	64,031	2,015,077.06	31.776	1.064 (1.054, 1.075)	30,536	12,490	198,253.1	63.0003	1.220 (1.196, 1.243)

Hazard ratios (HRs) and 95% confidence intervals (CIs) were estimated using the fully adjusted model (Model 4). Definitions of IR, HR, CI, Model 4, and reference group are provided in the footnote of Table 2. Smoking duration categories: <10, <20, ≥20 years; smoking amount: <10, <20, ≥20 cigarettes/day; pack-year: <10, <20, ≥20 pack-years. Incidence rates are crude (unadjusted), presented per 1000 person-years. Incident LSS was defined as ≥3 M48.06 claims on distinct service dates (outpatient or inpatient; same-day duplicates counted once; same provider not required); index date = date of the third claim.

### Appendix B.2. Association Between Smoking Burden and LSS by 10-Year Age Bands

This appendix provides extended age-stratified results using 10-year age bands (40–49, 50–59, 60–69, and ≥70 years), complementing the main findings reported in Section 3.3.1. Hazard ratios were estimated for each subgroup by smoking status and cumulative exposure, including duration, amount, and pack-years. The results demonstrate a progressive increase in risk with advancing age and higher smoking burden.

- Age 40–49: Significant risk was observed among current smokers with ≥20 years (HR 1.037), ≥20 cigarettes/day (HR 1.057), or ≥20 pack-years (HR 1.092). Among former smokers, only those with ≥20 pack-years showed increased risk (HR 1.058).
- Age 50–59: Risk was significantly elevated across most high-exposure categories. Former smokers with ≥20 pack-years had HR 1.102, while current smokers had HR 1.066.
- Age 60–69: Consistent risk elevation was seen in nearly all subgroups. The HR for former smokers with ≥20 pack-years (HR 1.139) slightly exceeded that of current smokers (HR 1.092).
- Age ≥ 70: This group exhibited the highest and most consistent risk. Current smokers with ≥20 years of smoking (HR 1.220), ≥20 cigarettes/day (HR 1.289), and ≥20 pack-years (HR 1.231) showed substantial increases, highlighting the amplified harm of continued smoking in older adults.

**Table A5:** Hazard ratios (HRs) and 95% confidence intervals (CIs) for LSS by combined smoking status and cumulative exposure, stratified by 10-year age bands (40–49, 50–59, 60–69, and ≥70 years).

Age Group	Category	N	Events	Duration (PY)	IR (per 1000 PY)	Model 4 HR (95% CI)
**40–49 years**	**Smoking status and Duration**					
	None	544,195	148,119	4,834,353.75	30.6388	1 (Ref.)
	Ex and <10	27,747	5762	255,757.17	22.5292	0.981 (0.956, 1.008)
	Ex and <20	61,787	12,800	570,044.06	22.4544	1.014 (0.995, 1.033)
	Ex and ≥20	47,760	10,836	435,473.06	24.8833	1.071 (1.050, 1.093)
	Current and <10	15,343	3668	138,058.69	26.5684	1.067 (1.032, 1.102)
	Current and <20	53,329	10,854	489,572.35	22.1704	0.998 (0.978, 1.018)
	Current and ≥20	177,925	37,421	1,626,795.59	23.0029	1.037 (1.024, 1.050)
	**Smoking status and amount**					
	None	544,195	148,119	4,834,353.75	30.6388	1 (Ref.)
	Ex and <10	17,126	3718	156,889.87	23.6982	1.005 (0.972, 1.038)
	Ex and <20	56,432	11,515	522,206.31	22.0507	0.992 (0.973, 1.012)
	Ex and ≥20	63,736	14,165	582,178.11	24.331	1.069 (1.050, 1.088)
	Current and <10	21,523	4818	195,612.48	24.6303	1.016 (0.987, 1.046)
	Current and <20	91,225	18,535	839,127.79	22.0884	1.002 (0.986, 1.018)
	Current and ≥20	133,849	28,590	1,219,686.35	23.4405	1.057 (1.043, 1.072)
	**Smoking status and Pack-year**					
	None	544,195	148,119	4,834,353.75	30.6388	1 (Ref.)
	Ex and <10	58,208	11,917	537,933.86	22.1533	0.984 (0.966, 1.004)
	Ex and <20	46,003	9721	423,526.72	22.9525	1.032 (1.010, 1.054)
	Ex and ≥20	33,083	7760	299,813.71	25.8827	1.107 (1.081, 1.133)
	Current and <10	50,274	10,887	458,211.87	23.7598	1.013 (0.993, 1.034)
	Current and <20	80,737	16,045	744,722.58	21.5449	0.998 (0.981, 1.015)
	Current and ≥20	115,586	25,011	1,051,492.19	23.7862	1.068 (1.052, 1.083)
**5** **0–** **5** **9 years**	**Smoking status and Duration**					
	None	411,790	164,725	3,317,386.08	49.6551	1 (Ref.)
	Ex and <10	14,921	4432	129,554.59	34.2095	0.943 (0.915, 0.972)
	Ex and <20	31,969	9569	276,704.22	34.5821	0.974 (0.953, 0.994)
	Ex and ≥20	65,953	20,981	561,416.34	37.3716	1.038 (1.022, 1.054)
	Current and <10	7148	2462	59,055.47	41.6896	1.052 (1.011, 1.095)
	Current and <20	14,946	4841	125,542.93	38.5605	1.014 (0.985, 1.044)
	Current and ≥20	124,195	38,072	1,049,336.88	36.282	1.035 (1.022, 1.047)
	**Smoking status and amount**					
	None	411,790	164,725	3,317,386.08	49.6551	1 (Ref.)
	Ex and <10	12,154	3662	105,270	34.7867	0.945 (0.915, 0.977)
	Ex and <20	41,873	12,302	363,566.82	33.837	0.959 (0.941, 0.978)
	Ex and ≥20	58,816	19,018	498,838.33	38.1246	1.057 (1.040, 1.074)
	Current and <10	11,973	3926	100,060.79	39.2361	1.006 (0.975, 1.039)
	Current and <20	50,194	14,968	428,380.7	34.9409	0.986 (0.969, 1.004)
	Current and ≥20	84,122	26,481	705,493.79	37.5354	1.070 (1.055, 1.085)
	**Smoking status and Pack-year**					
	None	411,790	164,725	3,317,386.08	49.6551	1 (Ref.)
	Ex and <10	33,881	10,031	294,240.46	34.0912	0.952 (0.932, 0.971)
	Ex and <20	32,561	9691	281,697.45	34.4022	0.976 (0.956, 0.997)
	Ex and ≥20	46,401	15,260	391,737.24	38.9547	1.075 (1.057, 1.094)
	Current and <10	21,548	7043	180,391.94	39.0428	1.009 (0.985, 1.034)
	Current and <20	34,730	10,295	296,804.67	34.6861	0.992 (0.972, 1.013)
	Current and ≥20	90,011	28,037	756,738.68	37.0498	1.060 (1.046, 1.075)
**6** **0–** **6** **9 years**	**Smoking status and Duration**					
	None	234,680	120,838	1,658,865.29	72.8438	1 (Ref.)
	Ex and <10	6032	2445	46,865.45	52.1706	0.983 (0.944, 1.023)
	Ex and <20	12,568	5109	97,522.43	52.3879	1.006 (0.977, 1.035)
	Ex and ≥20	45,100	18,962	339,119.86	55.9153	1.064 (1.047, 1.081)
	Current and <10	2054	880	15,145.61	58.1026	1.029 (0.963, 1.099)
	Current and <20	3546	1520	26,378.35	57.623	1.013 (0.963, 1.066)
	Current and ≥20	56,976	23,184	417,921.97	55.4745	1.077 (1.061, 1.093)
	**Smoking status and amount**					
	None	234,680	120,838	1,658,865.29	72.8438	1 (Ref.)
	Ex and <10	7810	3213	60,146.81	53.4193	0.997 (0.963, 1.033)
	Ex and <20	20,993	8507	162,021.57	52.5054	1.010 (0.987, 1.033)
	Ex and ≥20	34,897	14,796	261,339.37	56.616	1.079 (1.060, 1.098)
	Current and <10	7494	3136	54,468.42	57.5746	1.035 (0.999, 1.073)
	Current and <20	22,660	8983	168,821.56	53.21	1.024 (1.001, 1.046)
	Current and ≥20	32,422	13,465	236,155.95	57.0174	1.118 (1.098, 1.139)
	**Smoking status and Pack-year**					
	None	234,680	120,838	1,658,865.29	72.8438	1 (Ref.)
	Ex and <10	15,106	6126	117,395.9	52.1824	0.992 (0.966, 1.018)
	Ex and <20	15,537	6298	119,705.7	52.6124	1.013 (0.987, 1.040)
	Ex and ≥20	33,057	14,092	246,406.15	57.1901	1.088 (1.068, 1.108)
	Current and <10	7396	3165	54,281.03	58.3077	1.032 (0.996, 1.069)
	Current and <20	11,599	4659	86,469.59	53.8802	1.024 (0.994, 1.055)
	Current and ≥20	43,581	17,760	318,695.31	55.7272	1.095 (1.077, 1.114)
**≥70 years**	**Smoking status and Duration**					
	None	115,584	55,053	716,785.13	76.8054	1 (Ref.)
	Ex and <10	2031	849	13,318.97	63.7437	1.143 (1.068, 1.224)
	Ex and <20	3793	1581	24,850.39	63.6207	1.173 (1.115, 1.233)
	Ex and ≥20	20,254	8432	126,595.06	66.6061	1.234 (1.205, 1.263)
	Current and <10	473	197	2835.33	69.4805	1.142 (0.993, 1.314)
	Current and <20	666	265	4247.67	62.3871	1.010 (0.896, 1.140)
	Current and ≥20	20,503	8052	122,545.68	65.7061	1.220 (1.191, 1.249)
	**Smoking status and amount**					
	None	115,584	55,053	716,785.13	76.8054	1 (Ref.)
	Ex and <10	3575	1434	23,033.41	62.2574	1.107 (1.050, 1.167)
	Ex and <20	8819	3649	57,461.92	63.5029	1.177 (1.138, 1.218)
	Ex and ≥20	13,684	5779	84,269.1	68.5779	1.279 (1.244, 1.315)
	Current and <10	5421	2025	31,591.1	64.1003	1.086 (1.038, 1.135)
	Current and <20	8818	3456	53,665.08	64.3994	1.208 (1.167, 1.250)
	Current and ≥20	7403	3033	44,372.51	68.3531	1.319 (1.271, 1.368)
	**Smoking status and Pack-year**					
	None	115,584	55,053	716,785.13	76.8054	1 (Ref.)
	Ex and <10	5493	2251	36,035.84	62.4656	1.129 (1.082, 1.177)
	Ex and <20	5696	2385	36,891	64.6499	1.197 (1.149, 1.248)
	Ex and ≥20	14,889	6226	91,837.59	67.7936	1.265 (1.232, 1.299)
	Current and <10	2917	1153	17,375.78	66.3567	1.065 (1.004, 1.129)
	Current and <20	4327	1648	25,848.92	63.7551	1.137 (1.082, 1.194)
	Current and ≥20	14,398	5713	86,403.98	66.1196	1.272 (1.237, 1.308)

Hazard ratios (HRs) and 95% confidence intervals (CIs) were estimated using the fully adjusted Cox proportional hazards model (Model 4), adjusting for age, sex, body mass index, income level, alcohol consumption, regular exercise, diabetes mellitus, hypertension, and dyslipidemia. IR: incidence rate per 1000 person-years, calculated as the number of events divided by total follow-up time. Non-smokers served as the reference group (Ref.) in each exposure category. Smoking duration was categorized as <10, <20, and ≥20 years; smoking amount as <10, <20, and ≥20 cigarettes/day; and pack-year as <10, <20, and ≥20 pack-years. Incidence rates are crude (unadjusted), presented per 1000 person-years. Incident LSS was defined as ≥3 M48.06 claims on distinct service dates (outpatient or inpatient; same-day duplicates counted once; same provider not required); index date = date of the third claim.

### Appendix B.3. Sex-Stratified Association Between Combined Smoking Burden and LSS Risk

This section presents detailed numerical estimates for the sex-stratified analysis described in Section 3.3.2. The results illustrate marked differences between men and women in the association between smoking burden and the risk of developing LSS. In men, a history of prolonged and heavy smoking was associated with sustained elevated risk, even after cessation. Conversely, in women, current smoking was the dominant risk factor, with former smoking generally showing limited or no significant association. These findings suggest differing biological vulnerability and cessation-related reversibility between sexes. Full model-adjusted hazard ratios (Model 4) are provided in Table A6, with corresponding visualizations in the main text (Figure 12 and Figure 13).

**Table A6:** Sex-stratified hazard ratios for LSS according to combined smoking cessation status and cumulative exposure (Model 4).

	Male					Female				
	N	Event	Duration	IR, per 1000 PY	Model 4	N	Event	Duration	IR, per 1000 PY	Model 4
**Smoking status and Duration**										
None	357,217	104,334	3,007,774.09	34.6881	1 (Ref.)	949,032	384,401	7,519,616.15	51.1198	1 (Ref.)
Ex and <10	44,935	11,429	397,632.64	28.7426	0.960 (0.942, 0.979)	5796	2059	47,863.55	43.0181	1.014 (0.971, 1.059)
Ex and <20	106,652	27,717	941,295.46	29.4456	0.993 (0.980, 1.006)	3465	1342	27,825.64	48.2289	1.038 (0.984, 1.095)
Ex and ≥20	177,044	58,258	1,448,421.49	40.2217	1.080 (1.069, 1.091)	2023	953	14,182.83	67.1939	0.995 (0.934, 1.061)
Current and <10	17,873	4322	157,919.47	27.3684	0.973 (0.944, 1.003)	7145	2885	57,175.63	50.4586	1.191 (1.148, 1.235)
Current and <20	62,262	13,272	564,953.24	23.4922	0.931 (0.914, 0.948)	10,225	4208	80,788.06	52.0869	1.182 (1.147, 1.219)
Current and ≥20	369,175	101,767	3,142,293.01	32.3862	1.049 (1.040, 1.059)	10,424	4962	74,307.12	66.7769	1.129 (1.097, 1.161)
**Smoking status and amount**										
None	357,217	104,334	3,007,774.09	34.6881	1 (Ref.)	949,032	384,401	7,519,616.15	51.1198	1 (Ref.)
Ex and <10	35,010	9971	299,350.64	33.3088	0.989 (0.969, 1.010)	5655	2056	45,989.44	44.7059	0.979 (0.937, 1.022)
Ex and <20	124,059	34,380	1,072,986.15	32.0414	0.992 (0.979, 1.004)	4058	1593	32,270.46	49.364	1.039 (0.989, 1.092)
Ex and ≥20	169,562	53,053	1,415,012.78	37.4929	1.084 (1.072, 1.095)	1571	705	11,612.12	60.7124	1.091 (1.013, 1.174)
Current and <10	35,854	9639	299,524.24	32.181	0.984 (0.963, 1.005)	10,557	4266	82,208.55	51.8924	1.063 (1.032, 1.096)
Current and <20	160,916	40,680	1,398,816.79	29.0817	0.983 (0.972, 0.995)	11,981	5262	91,178.35	57.7111	1.204 (1.172, 1.237)
Current and ≥20	252,540	69,042	2,166,824.69	31.8632	1.073 (1.063, 1.084)	5256	2527	38,883.92	64.9883	1.266 (1.217, 1.316)
**Smoking status and Pack-year**										
None	357,217	104,334	3,007,774.09	34.6881	1 (Ref.)	949,032	384,401	7,519,616.15	51.1198	1 (Ref.)
Ex and <10	103,602	26,995	911,509.23	29.6157	0.970 (0.957, 0.983)	9086	3330	74,096.83	44.9412	1.004 (0.970, 1.039)
Ex and <20	98,397	27,458	851,443.59	32.2488	1.009 (0.996, 1.023)	1400	637	10,377.28	61.3841	1.097 (1.015, 1.186)
Ex and ≥20	126,632	42,951	1,024,396.76	41.9281	1.109 (1.097, 1.122)	798	387	5397.92	71.6943	1.006 (0.911, 1.112)
Current and <10	64,347	15,015	570,003.8	26.3419	0.947 (0.930, 0.963)	17,788	7233	140,256.82	51.5697	1.137 (1.111, 1.164)
Current and <20	125,045	29,671	1,107,185.19	26.7986	0.970 (0.957, 0.983)	6348	2976	46,660.57	63.7798	1.204 (1.161, 1.248)
Current and ≥20	259,918	74,675	2,187,976.73	34.1297	1.079 (1.069, 1.090)	3658	1846	25,353.42	72.8107	1.195 (1.142, 1.251)

Hazard ratios (HRs) and 95% confidence intervals (CIs) were estimated using the fully adjusted model (Model 4). Definitions of IR, HR, CI, Model 4, and reference group are provided in the footnote of Table 2. Smoking duration categories: <10, <20, ≥20 years; smoking amount: <10, <20, ≥20 cigarettes/day; pack-year: <10, <20, ≥20 pack-years. Results are stratified by sex (male, female). Incidence rates are crude (unadjusted), presented per 1000 person-years. Incident LSS was defined as ≥3 M48.06 claims on distinct service dates (outpatient or inpatient; same-day duplicates counted once; same provider not required); index date = date of the third claim.

## Appendix C

### Appendix C.1. Subgroup and Interaction Analyses for Smoking-Related Risk of LSS

This appendix provides detailed subgroup-specific hazard ratios and *p*-values for interaction terms that examine whether the association between smoking and LSS differed across demographic and clinical subpopulations. As described in Section 3.4, significant effect modifications were observed for age and sex, with higher relative risks seen in women and older adults. Other modifiers such as income, obesity, hypertension, alcohol use, and exercise habits also influenced the strength of association. The full results are presented in Table A7. Visual summaries are provided in the main text (Figure 14), including both the original and sorted views of subgroup hazard ratios for current smokers.

**Table A7:** Subgroup and interaction analyses of the association between smoking and risk of LSS (Model 4).

		Smoking	N	Event	Duration	IR, per 1000 PY	HR (95% C.I)	*p* for Interaction
**Age groups**	40–64	None	1,099,107	384,974	9,190,028.6	41.8904	1 (Ref.)	<0.0001
		Former	289,693	80,465	2,537,191.47	31.7142	1.016 (1.007, 1.025)	
		Current	432,328	113,120	3,787,142.02	29.8695	1.027 (1.019, 1.036)	
	≥65	None	207,142	103,761	1,337,361.64	77.5863	1 (Ref.)	
		Former	50,222	21,293	340,030.13	62.6209	1.146 (1.129, 1.164)	
		Current	44,776	18,296	290,294.51	63.0256	1.174 (1.155, 1.193)	
**Sex**	Male	None	357,217	104,334	3,007,774.09	34.6881	1 (Ref.)	<0.0001
		Former	328,631	97,404	2,787,349.58	34.945	1.039 (1.030, 1.048)	
		Current	449,310	119,361	3,865,165.72	30.8812	1.032 (1.024, 1.041)	
	Female	None	949,032	384,401	7,519,616.15	51.1198	1 (Ref.)	
		Former	11,284	4354	89,872.02	48.4467	1.017 (0.987, 1.048)	
		Current	27,794	12,055	212,270.81	56.7907	1.161 (1.140, 1.183)	
**Income**	Q2–4	None	1,012,450	372,930	8,188,085.61	45.5454	1 (Ref.)	0.0005
		Former	289,948	84,875	2,471,224.75	34.3453	1.051 (1.042, 1.060)	
		Current	389,716	104,906	3,356,176.65	31.2576	1.058 (1.049, 1.067)	
	Q1	None	293,799	115,805	2,339,304.63	49.504	1 (Ref.)	
		Former	49,967	16,883	405,996.85	41.5841	1.032 (1.015, 1.050)	
		Current	87,388	26,510	721,259.88	36.7551	1.029 (1.015, 1.044)	
**Obesity BMI ≥ 25**	No	None	888,363	311,645	7,285,125.45	42.7783	1 (Ref.)	<0.0001
		Former	201,186	57,927	1,706,108.62	33.9527	1.064 (1.053, 1.075)	
		Current	315,768	85,234	2,690,884.35	31.6751	1.084 (1.074, 1.094)	
	Yes	None	417,886	177,090	3,242,264.79	54.6192	1 (Ref.)	
		Former	138,729	43,831	1,171,112.99	37.4268	1.023 (1.011, 1.035)	
		Current	161,336	46,182	1,386,552.18	33.3071	0.997 (0.986, 1.009)	
**Abdominal obesity WC ≥ 90/85**	No	None	1,050,019	373,418	8,611,942.99	43.3605	1 (Ref.)	<0.0001
		Former	254,634	72,921	2,176,702.42	33.5007	1.050 (1.040, 1.059)	
		Current	375,478	100,325	3,224,739.94	31.111	1.061 (1.052, 1.071)	
	Yes	None	256,230	115,317	1,915,447.25	60.2037	1 (Ref.)	
		Former	85,281	28,837	700,519.18	41.1652	1.039 (1.025, 1.054)	
		Current	101,626	31,091	852,696.59	36.462	1.020 (1.006, 1.033)	
**Heavy Drinking**	No	None	1,274,037	477,654	10,263,653.04	46.5384	1 (Ref.)	<0.0001
		Former	294,203	87,686	2,489,838.14	35.2176	1.052 (1.043, 1.062)	
		Current	388,734	106,440	3,328,505.56	31.9783	1.057 (1.049, 1.066)	
	Yes	None	32,212	11,081	263,737.2	42.0153	1 (Ref.)	
		Former	45,712	14,072	387,383.46	36.3258	0.998 (0.974, 1.023)	
		Current	88,370	24,976	748,930.97	33.3489	1.004 (0.982, 1.027)	
**Regular exercise**	No	None	1,056,687	394,391	8,510,009.22	46.3444	1 (Ref.)	0.0006
		Former	247,026	73,379	2,091,526.47	35.0839	1.055 (1.046, 1.065)	
		Current	390,873	106,624	3,346,129.42	31.8649	1.052 (1.043, 1.060)	
	Yes	None	249,562	94,344	2,017,381.02	46.7656	1 (Ref.)	
		Former	92,889	28,379	785,695.13	36.1196	1.025 (1.011, 1.040)	
		Current	86,231	24,792	731,307.12	33.9009	1.055 (1.039, 1.070)	
**Diabetes mellitus**	No	None	1,182,850	436,748	9,617,386.13	45.4123	1 (Ref.)	0.6053
		Former	293,360	86,456	2,504,674.67	34.5179	1.047 (1.038, 1.056)	
		Current	414,622	112,825	3,571,551.9	31.5899	1.051 (1.042, 1.059)	
	Yes	None	123,399	51,987	910,004.11	57.1283	1 (Ref.)	
		Former	46,555	15,302	372,546.93	41.074	1.050 (1.031, 1.070)	
		Current	62,482	18,591	505,884.64	36.7495	1.060 (1.042, 1.079)	
**Hypertension**	No	None	915,672	320,598	7,602,004.07	42.1728	1 (Ref.)	<0.0001
		Former	214,932	60,003	1,865,175.94	32.1702	1.039 (1.029, 1.050)	
		Current	336,565	88,176	2,931,440.05	30.0794	1.043 (1.033, 1.052)	
	Yes	None	390,577	168,137	2,925,386.18	57.4751	1 (Ref.)	
		Former	124,983	41,755	1,012,045.67	41.258	1.059 (1.047, 1.072)	
		Current	140,539	43,240	1,145,996.49	37.7314	1.071 (1.058, 1.083)	
**Dyslipidemia**	No	None	1,029,585	368,292	8,421,523.44	43.7322	1 (Ref.)	0.9503
		Former	263,858	77,121	2,247,188.26	34.3189	1.048 (1.038, 1.058)	
		Current	383,004	103,183	3,287,469.18	31.3868	1.052 (1.043, 1.061)	
	Yes	None	276,664	120,443	2,105,866.8	57.194	1 (Ref.)	
		Former	76,057	24,637	630,033.34	39.1043	1.045 (1.030, 1.061)	
		Current	94,100	28,233	789,967.36	35.7395	1.052 (1.037, 1.066)	

HRs were derived from the fully adjusted Cox proportional hazards model (Model 4), adjusting for age, sex, body mass index, income level, alcohol consumption, regular exercise, diabetes mellitus, hypertension, and dyslipidemia. p for interaction was calculated to assess whether the association between smoking and LSS differed significantly across subgroups. P for interaction values were derived from multiplicative interaction terms in the Cox proportional hazards models.

## Appendix D

### Standardized Mean Differences (SMDs) for Baseline Characteristics

To supplement Table 1, we present standardized mean differences (SMDs) between smoking status groups (never, former, and current smokers). SMDs were calculated for continuous variables as the mean difference between groups divided by the pooled standard deviation. An absolute SMD greater than 0.1 was considered indicative of meaningful imbalance between groups.

**Table A8:** Standardized mean differences (SMDs) for baseline characteristics among smoking groups.

	Non vs. Former	Non vs. Current	Former vs. Current
**Age, years**	0.00	0.25	0.27
**Height, cm**	−1.13	−1.18	−0.06
**Weight, kg**	−0.92	−0.72	0.18
**BMI, kg/m** **^2^**	−0.21	−0.01	0.2
**Waist Circumference, cm**	−0.66	−0.49	0.18
**Systolic BP, mmHg**	−0.21	−0.11	0.10
**Diastolic BP, mmHg**	−0.26	−0.2	0.06
**Fasting glucose, mg/dL**	−0.19	−0.17	0.01
**Total Cholesterol, mg/dL**	0.02	0.01	−0.02
**HDL-C, mg/dL**	0.13	0.15	0.02
**LDL-C, mg/dL**	0.08	0.14	0.06
**Triglyceride, mg/dL**	−0.21	−0.31	−0.09

Values represent standardized mean differences (SMDs) between smoking status groups. An absolute SMD > 0.1 indicates a potentially meaningful imbalance. Abbreviations: BMI, body mass index; BP, blood pressure; HDL-C, high-density lipoprotein cholesterol; LDL-C, low-density lipoprotein cholesterol.
